# Muscular Assessment in Patients With Severe Obstructive Sleep Apnea Syndrome: Protocol for a Case-Control Study

**DOI:** 10.2196/30500

**Published:** 2021-08-06

**Authors:** Paz Francisca Borrmann, Carlos O'Connor-Reina, Jose M Ignacio, Elisa Rodriguez Ruiz, Laura Rodriguez Alcala, Florencia Dzembrovsky, Peter Baptista, Maria T Garcia Iriarte, Carlos Casado Alba, Guillermo Plaza

**Affiliations:** 1 Phonoaudiology Section Otorhinolaryngology Department Hospital Italiano de Buenos Aires Buenos Aires Argentina; 2 Otorhinolaryngology Department Hospital Quironsalud Marbella Marbella (Malaga) Spain; 3 Otorhinolaryngology Department Hospital Quironsalud Campo de Gibraltar Palmones, Cadiz Spain; 4 Pulmonology Department Hospital Quironsalud Marbella Malaga Spain; 5 Pulmonology Department Hospital Quironsalud Campo de Gibraltar Palmones, Cadiz Spain; 6 Otorhinolaryngology Department Clinica Universitaria de Navarra Pamplona Spain; 7 Otorhinolaryngology Department Hospital Universitario Virgen de Valme Sevilla Spain; 8 School of Medicine Clinica Universitaria de Navarra Pamplona Spain; 9 Otorhinolaryngology Department Hospital Universitario Sanitas la Zarzuela Madrid Spain; 10 Otorhinolaryngology Department Hospital Universitario Fuenlabrada Madrid Spain; 11 School of Medicine Universidad Rey Juan Carlos I Madrid Spain

**Keywords:** myofunctional therapy, sleep apnea, sleep disordered breathing, speech therapy, phenotype, sleep, therapy, protocol, muscle, assessment, case study, exercise, airway, respiratory

## Abstract

**Background:**

Myofunctional therapy is currently a reasonable therapeutic option to treat obstructive sleep apnea-hypopnea syndrome (OSAHS). This therapy is based on performing regular exercises of the upper airway muscles to increase their tone and prevent their collapse. Over the past decade, there has been an increasing number of publications in this area; however, to our knowledge, there are no studies focused on patients who can most benefit from this therapy.

**Objective:**

This protocol describes a case-control clinical trial aimed at determining the muscular features of patients recently diagnosed with severe OSAHS compared with those of healthy controls.

**Methods:**

Patients meeting set criteria will be sequentially enrolled up to a sample size of 40. Twenty patients who meet the inclusion criteria for controls will also be evaluated. Patients will be examined by a qualified phonoaudiologist who will take biometric measurements and administer the Expanded Protocol of Orofacial Myofunctional Evaluation with Scores (OMES), Friedman Staging System, Epworth Sleepiness Scale, and Pittsburgh Sleep Quality Index questionnaires. Measures of upper airway muscle tone will also be performed using the Iowa Oral Performance Instrument and tongue digital spoon devices. Evaluation will be recorded and reevaluated by a second specialist to determine concordance between observers.

**Results:**

A total of 60 patients will be enrolled. Both the group with severe OSAHS (40 patients) and the control group (20 subjects) will be assessed for differences between upper airway muscle tone and OMES questionnaire responses.

**Conclusions:**

This study will help to determine muscle patterns in patients with severe OSAHS and can be used to fill the gap currently present in the assessment of patients suitable to be treated with myofunctional therapy.

**Trial Registration:**

ISRCTN Registry ISRCTN12596010; https://www.isrctn.com/ISRCTN12596010

**International Registered Report Identifier (IRRID):**

PRR1-10.2196/30500

## Introduction

### Background

Obstructive sleep apnea-hypopnea syndrome (OSAHS) is a significant public health issue characterized by repetitive episodes of airway obstruction during sleep associated with snoring, sleep fragmentation, daytime sleepiness, and increased cardiovascular risk [1,2]. It is well established that the most effective treatment for OSAHS is continuous positive airway pressure (CPAP) [3], which has variable patient compliance. CPAP virtually eliminates OSAHS and snoring, reduces daytime sleepiness, and improves subjective sleep quality [3,4].

The etiology of OSAHS is multifactorial, including anatomical and physiological factors. The upper airway dilator muscles are crucial for maintaining pharyngeal patency and may contribute to the incidence of this medical condition [5,6].

Other treatments for OSAHS include a mandibular advancement device (MAD), conventional surgery, CO_2_ or radiofrequency laser, or hypoglossal nerve stimulation. There is also some evidence for pharmacological treatments with oxybutynin and atomoxetine, which are currently showing promising results [7]. Clinical trials have been carried out with theophylline, acetazolamide, and desipramine to reduce the collapse of the upper airway, but without clear effectiveness [8,9].

Myofunctional therapy is a treatment applied to patients with orofacial myofunctional disorders that can interfere with the development or functioning of orofacial structures and functions [10]. Reviews of studies on myofunctional therapy show benefits by promoting changes in dysfunctional muscles of the upper airway [11]; therefore, this treatment has been proposed to reduce the severity of OSAHS and associated symptoms in adults [12]. Myofunctional therapy also has potential to promote a decrease in the Apnea-Hypopnea Index (AHI), reduce snoring [13], and improve quality of life [14]. In addition, it can be considered as an adjuvant therapy and an intervention strategy to support CPAP adherence [15].

However, it is currently unknown which patients are the best candidates for myofunctional therapy. Although there are instruments available for patient selection such as the Expanded Protocol of Orofacial Myofunctional Evaluation with Scores (OMES), involving functional exploration of all of the stomatognathic functions to obtain a score, this has proven to be inferior in patients with OSAHS compared with controls [16,17]. A myofunctional therapist uses this evaluation to improve the examined items that are in deficit and subsequently performs specific exercises to improve them. However, the OMES test is based on subjective evaluations, contains many items, and is difficult to reproduce. In our opinion, a more concise, objective, and reproducible evaluation is required. This opinion stems from our experience of measurements with the Iowa Oral Performance Instrument (IOPI) of the genioglossus muscle and the orbicular muscle [18,19]. Together with measurement of the motor tone of the genioglossus muscle using a tongue digital spoon (TDS) [20], these simple measurements may provide patients with information about their condition, serve as therapy response parameters, and objectively transmit results between professionals, which can also be based in electronic health facilities [21].

### Aim

The aim of this study is to evaluate the muscle patterns of patients with severe OSAHS. The use of the OMES protocol can be complemented by the values obtained through the IOPI and the TDS.

### Objectives

The main objective is to evaluate the function of the stomatognathic musculature of patients with OSAHS by using the OMES protocol, TDS, and IOPI.

The secondary objectives are to: (1) use this protocol to evaluate whether there are differences between the muscles of patients with OSAHS and healthy controls; (2) use the IOPI to measure tongue strength and resistance with the genioglossus and buccinator muscle tone, and evaluate whether there are differences from those of healthy patients; and (3) use the TDS to measure tongue pressure and evaluate whether there are differences from healthy patients.

## Methods

### Design

**Figure 1:**
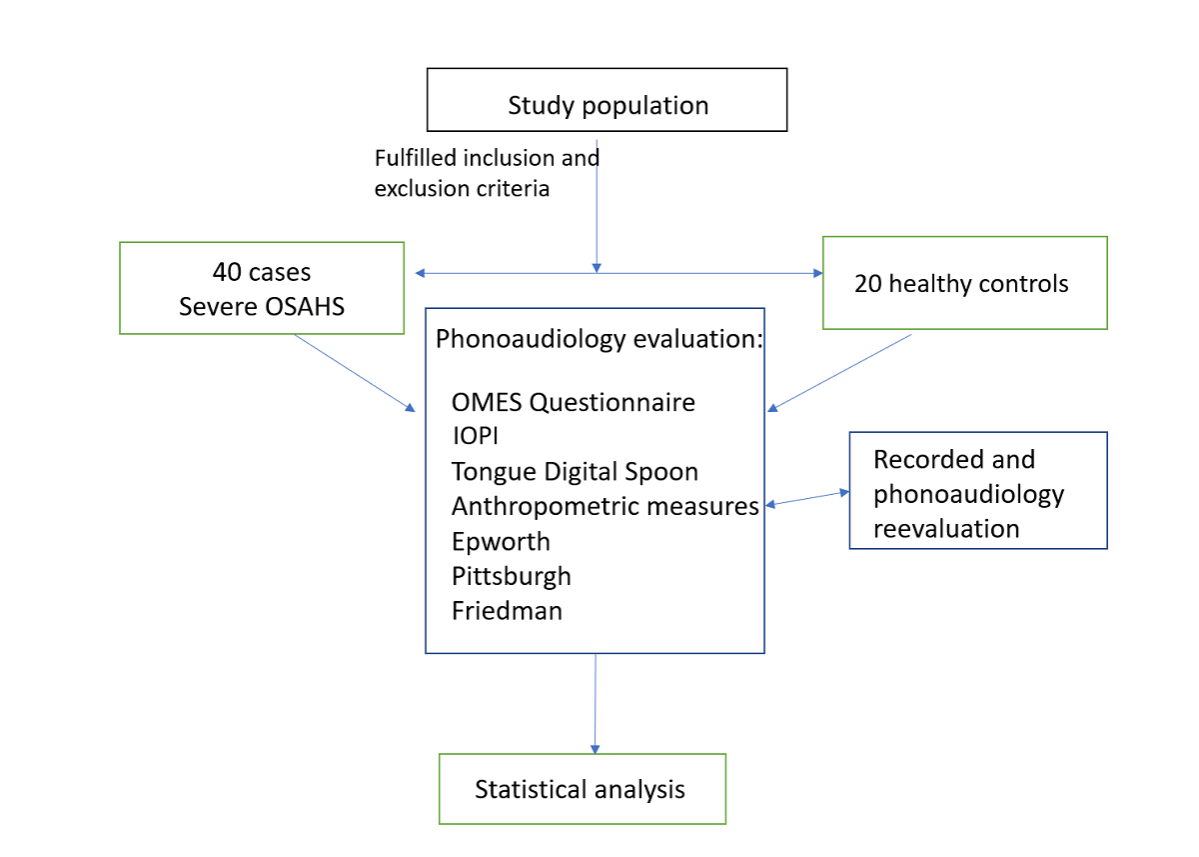
Flow chart of the study process. IOPI: Iowa Oral Performance Instrument; OMES: Orofacial Myofunctional Evaluation with Scores; OSAHS: obstructive sleep apnea-hypopnea syndrome; TDS: tongue digital spoon.

We designed a prospective controlled quasiexperimental pilot study on patients with severe OSAHS (Figure 1).

### Scope of the Study

This study will involve patients diagnosed and/or treated at the Pneumology and Otorhinolaryngology Departments of Quirónsalud Hospital in Marbella (Malaga, Spain) or Hospital Campo de Gibraltar (Palmones, Cadiz, Spain) where the study will also be performed.

### Study Population

This study will include patients diagnosed with sleep apnea-hypopnea at the participating hospitals and who agree to participate in the project.

### Inclusion Criteria

For cases, adults (aged 18-75 years) who have received a diagnosis of severe OSAHS (AHI>30) without previous experience of the condition and not undergoing treatment owing to different circumstances, who had not undergone any previous treatment for OSAHS, and signed the informed consent form will be included.

For controls, adults with adequate sleep hygiene, no complaints of snoring or daytime sleepiness, and scoring <7 points on the Epworth Sleepiness Scale will be included.

### Exclusion Criteria

Cases and controls alike with a cognitive or neurological deficit, inability to answer questionnaires, severe alcohol abuse, presence of craniofacial malformations, active neoplastic disease, or a history of prior orofacial muscle rehabilitation therapy and any prior apnea treatment that may modify the study results (surgery, MAD, CPAP) will be excluded.

### Sample Size and Sampling Procedure

The effectiveness of use of the OMES protocol in the evaluation of patients with moderate to severe apnea-hypopnea syndrome will be evaluated from data previously published in studies using this protocol. Following the literature review, patients will be recruited. The sample size will be 60 subjects (40 in the experimental group and 20 in the control group). The sample size was calculated using the XLSTAT statistical software for Excel.

The variables that we are going to measure in all patients are reflected in the data collection table shown in Multimedia Appendix 1 using SPSS Info software, including age, sex, weight, height, ethnicity, BMI, abdominal circumference (at the level of the navel), neck circumference (using a flexible tape around the most prominent part while the patient is standing with their arms by their sides, head erect, and eyes looking ahead), IOPI measurement of tongue strength and the buccinator muscle, AHI, nighttime oxygen desaturation index, lowest overnight oxygen saturation levels, digital spoon measurement of tongue strength, and OMES protocol.

A series of questionnaires will be applied to both groups: Friedman Staging System, Epworth Sleepiness Scale, and Pittsburgh Sleep Quality Index (see Multimedia Appendix 2). An information sheet and information consent will also be provided and signed by patients.

### Procedures

#### Experimental Design

A myofunctional evaluation of patients diagnosed with OSAHS will be performed in the same week as the polysomnography is performed. During this evaluation, patients will be blindly examined by a speech therapist and their examination will be recorded on video for subsequent evaluation.

The patient will sit 1 meter away from the camera with their feet flat on the floor and their back supported by the backrest. The camera (Sony CCD-TRV138 Handycam camcorder) will be placed on a tripod at face and shoulder height.

Evaluation with the OMES protocol (see Multimedia Appendix 3) will then take place, based on the analysis of the following parameters: (1) appearance/posture; (2) mobility; and (3) functions, including respiration, deglutition, and mastication.

As a result of this evaluation with the already validated protocol, the higher the score, the more normal the patient’s stomatognathic system.

#### IOPI Evaluation

The IOPI objectively measures maximum tongue and lip strength. Tongue strength is assessed by measuring the maximum pressure exerted when a person presses a disposable, standard-sized tongue bulb against the roof of the mouth. Lip strength is assessed by measuring the maximum pressure when the bulb is located between the cheek and closed teeth, and the patient contracts the buccinator muscle without biting the bulb. Reference values have been obtained for a healthy population and are provided by the manufacturer [18].

Tongue strength is measured by obtaining maximal tongue elevation pressures. The patient is instructed to “place this bulb in your mouth on the midline of your tongue and push it against the roof of your mouth as hard as you can.” To maximize standard placement, the examiner demonstrates how to place the bulb along the central groove of the tongue blade. Previous research [18] indicates that maximal measures of tongue strength and endurance are best assessed with an unconstrained jaw; participants will be encouraged to gently rest the incisors on the tubing of the IOPI bulb. Each test lasts 7-10 seconds, and all participants will be given verbal encouragement from the examiner for the entire test. The test will be performed three times by each participant, with a brief rest of about 30 seconds between each test while the examiner records the peak pressure obtained. The highest pressure across the three trials will be used as the maximal isometric pressure instead of the mean pressure, which has been used by other researchers [22]. Given the high correlation between the average and maximal pressure and that both are similarly related to oral-phase swallowing function, the use of maximal pressure is more efficient in a clinical setting because it requires no calculation.

Subsequently, the muscle tone of the genioglossus muscle and the buccinator muscle are evaluated, taking three measurements of each and using the highest value.

#### TDS Evaluation

Finally, the tone of the tongue muscles will be measured with a digital spoon, taking three measurements and using the highest obtained. A digital spoon is a kitchen tool used to estimate the weight of food. To develop the TDS, we used the Soehnle Cooking Star Digital Measuring Spoon with graduation from 0.1 grams to 500 grams (ID ID20005876833). This is a handheld instrument with a spoon that can be found on online shopping platforms, consisting of a handle where the “tare” and “hold” buttons are located. Pressing the “hold” button helps to obtain the highest tared value, equivalent to the IOPI peak pressure. To carry out the measurements, the spoon is inverted and a 1-cm^2^ circular sticker is placed on the back to obtain a marked circumference. To measure tongue strength, the subject holds the spoon by the handle and, with their elbow resting on a flat surface, brings the spoon closer to the tongue with an elbow angle of approximately 30°. The subject must then tare the device by pressing the “hold” key, marking 0.0 grams. The subject then presses on the marked circumference with the tip of their tongue. Once done, with the index finger of the hand that is holding the handle, the subject presses the “hold” button. This test is performed entirely by the subject to avoid movements on the spoon that may interfere with the results [20].

The recordings and the data obtained will also be analyzed by another blinded examiner.

### Distribution of Hospital Visits

#### Selection Visit

A patient diagnosed with OSAHS at a pulmonology laboratory by means of an initial sleep study (with measurement of baseline AHI, nighttime oxygen desaturation index, and the lowest nighttime oxygen saturation figures) will be evaluated with respect to the inclusion and exclusion criteria and then informed about the study. After reading the information and having any doubts resolved, the patient will accept and sign the inform consent form in duplicate, taking one copy home.

#### One-off Visit

The patient will be evaluated by the speech therapist and fill in the sleepiness questionnaires, following which the OMES protocol will be applied and the evaluation will be carried out with the IOPI and the TDS.

### Statistical Analysis

The data of the study variables will be collected in a database created for the development of the study. In the statistical analysis, the sample will be described through the distribution of frequencies for the categorical variables, and through measures of central tendency and dispersion such as the mean (SD) and median (IQR) for quantitative variables. The distribution of quantitative variables will be examined using the Kolmogorov-Smirnov test. Bivariate analysis of the association between categorical variables will be carried out using the χ^2^ test or Fisher exact test when necessary. The differences between quantitative variables will be analyzed using the Student *t* test or analysis of variance for two or more samples, respectively, and nonparametric tests (Mann-Whitney or Kruskal-Wallis test) will be used if the variables to be analyzed do not follow the normal distribution. The possible correlations between the OMES protocol evaluation and the IOPI values and TDS will be assessed using the Spearman rank correlation coefficient. The consistency and stability of the intra- and interrater measurements (reliability coefficient) will be determined using the split-half method. The level of statistical significance will be set at *P*<.05.

### Ethical Aspects

The Research Ethics Committee of the Hospital Provincial de Málaga reviewed and approved the protocol and the informed consent model for the patients (AWGAP-2021-02). Before performing any of the procedures specified in the study protocol, the participating subjects will have signed and dated the informed consent form approved by the Research Ethics Committee.

### Access to Data and Protection of Data Obtained from the Study

To guarantee the confidentiality of the study data, the original data will be stored at the hospital and only researchers and the Research Ethics Committee will have access.

This project will be carried out following the guidelines of the Declaration of Helsinki (Fortaleza, Brazil, 2013) [23] and the Standards of Good Clinical Practice. Personal data will be processed according to Regulation (EU) 2016/679 of the European Parliament and of the Council (April 27, 2016) on the protection of natural persons with regard to the processing of personal data and on the free movement of such data, and Organic Law 3 (December 5, 2018) on the protection of personal data and guarantee of digital rights.

### Usefulness and Applicability

The selection criteria for patients with OSAHS may improve depending on which therapy is more suitable.

## Results

**Figure 2:**
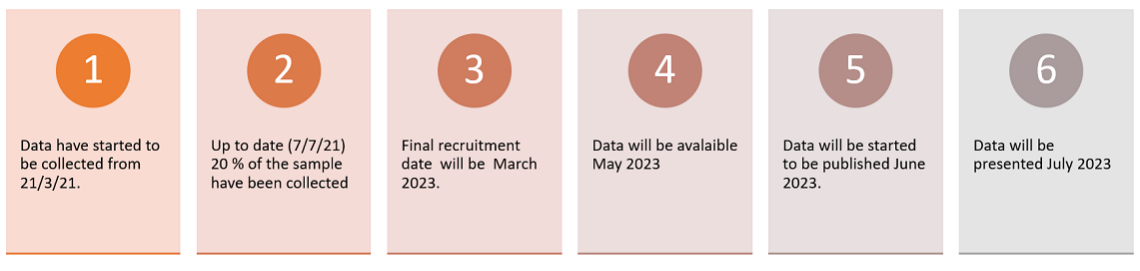
Summary of the results.

The authors plan to publish the study findings in a peer-reviewed journal and at topic-related conferences (to be determined at a later date). All listed authors or contributors are compliant with guidelines outlined by the International Committee of Medical Journal Editors for author inclusion in a published work. Furthermore, to support research transparency and reproducibility, we will share the deidentified research data after publication of the study results. We will also share the deidentified data on Figshare, a repository where users can make all of their research outputs available in a citable, shareable, and discoverable manner. To date, we have collected data for 20% of the planned sample. The timeline for data collection to completion of the study is given in Figure 2.

## Discussion

### Projected Significance

Although Eckert [24] proposed that one of the phenotypes responsible for initiating sleep apnea is the hypotonic pattern in 2016, studies have yet to be performed to confirm this proposal. It is well known that patients with this phenotype can benefit from treatment with a hypoglossal pacemaker, myotonic medication such as desipramine, and myofunctional therapy. We consider that our study will help to identify certain patients with severe OSAHS according to distinct anthropometric and myofunctional features from those of conventional patients (ie, individuals with obesity or with anatomical issues such as big tonsils). Following our experience using the IOPI [18], we consider that patients with a normal BMI, neck and bell circumference, and anatomy of the upper airway will show no relationship with the position of the tongue or the soft palate. We previously demonstrated that Friedman stage is independent from the tone of the muscle of the genioglossus as measured by the IOPI [18].

The patients’ main anomaly will be determined by their responses to the OMES questionnaire, and with the measures of the upper airway muscles using the IOPI and TDS.

The TDS is a simple, reproducible, and affordable method to measure the muscle tone of the tongue for this patient group. We have pioneered the use of this domestic tool to allow patients to obtain immediate feedback of their success in performing myofunctional therapy exercises. In our opinion, the OMES questionnaire is a suitable tool to make a diagnosis for these patients, but can only be performed by specialized phonoaudiologists and requires considerable time. In most countries, there is a lack of phonoaudiologists and the demand on their time means that consultations are short. We contend that we can provide this information with the assistance of the IOPI and TDS that do not require any special training.

The information provided by these two instruments can be correlated with the information obtained by the OMES. In this case, these two measures are fast, simple, reproducible, and provide objective information to both the patient and examiner.

### Limitations

One of the limitations of this study is that although we are going to identify patients according to their singular characteristics, we are not going to be able to demonstrate the effectiveness of the exercises performed with myofunctional therapy. Theoretically, these patients should improve with myofunctional therapy, a hypoglossal pacemaker, or inotropic medication. We intend to perform that study as a continuation of this proposed trial.

Our main concern is that if these patients have myofunctional disorders (ie, low OMES, IOPI, or TDS scores), the therapist is obliged to correct the deficits simultaneously with the use of CPAP. We are strongly opposed to any surgery in patients where there is a demonstrated myofunctional disorder.

### Comparison With Prior Work

No previous work directed at this matter has been performed for effective comparison.

### Conclusions

This study will help determine the muscle patterns in patients with severe OSAHS and may be used to fill the current gap in the identification of patients suitable to be treated with myofunctional therapy.

## Appendix

Multimedia Appendix 1 PowerPoint presentation with database.

Multimedia Appendix 2 Information sheets; informed consent; and Pittsburg, Friedman, and Epworth questionnaires.

Multimedia Appendix 3 OMES questionnaire.

## Abbreviations

AHI: Apnea-Hypopnea Index
CPAP: continuous positive airway pressure
IOPI: Iowa Oral Performance Instrument
MAD: mandibular advancement device.
OMES: Orofacial Myofunctional Evaluation with Scores
OSAHS: obstructive sleep apnea-hypopnea syndrome.
TDS: tongue digital spoon
