# Supplementary material for: Muscular Assessment in Patients With Severe Obstructive Sleep Apnea Syndrome: Protocol for a Case-Control Study
Source: JMIR Res Protoc. 2021 Aug 6;10(8):e30500. doi: 10.2196/30500 (PMC8380583; doi:10.2196/30500)
Supplement: Multimedia Appendix 1 [file resprot_v10i8e30500_app1.pptx]

## Slide 1
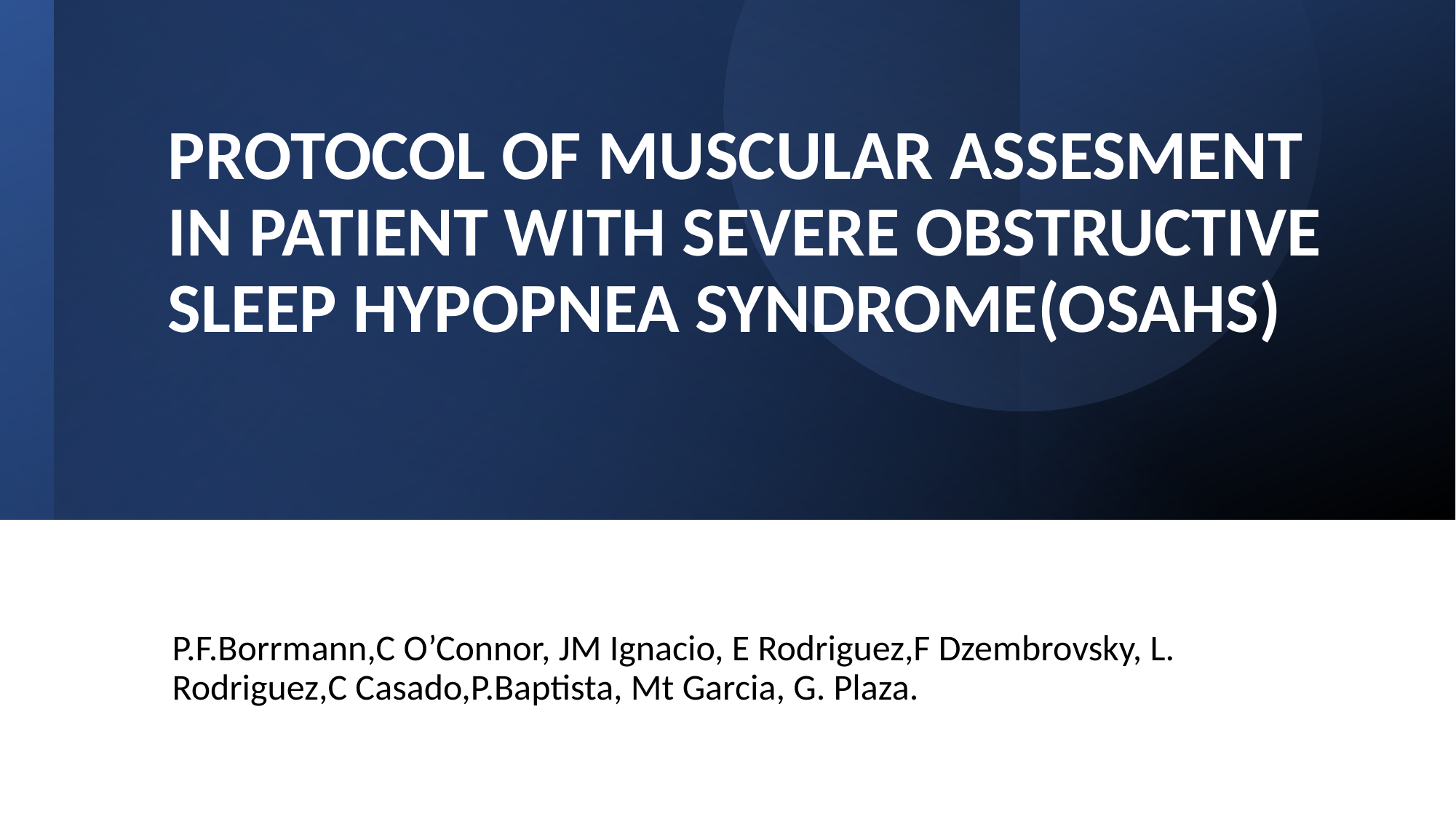

# PROTOCOL OF MUSCULAR ASSESMENT IN PATIENT WITH SEVERE OBSTRUCTIVE SLEEP HYPOPNEA SYNDROME(OSAHS)
P.F.Borrmann,C O’Connor, JM Ignacio, E Rodriguez,F Dzembrovsky, L. Rodriguez,C Casado,P.Baptista, Mt Garcia, G. Plaza.

## Slide 2
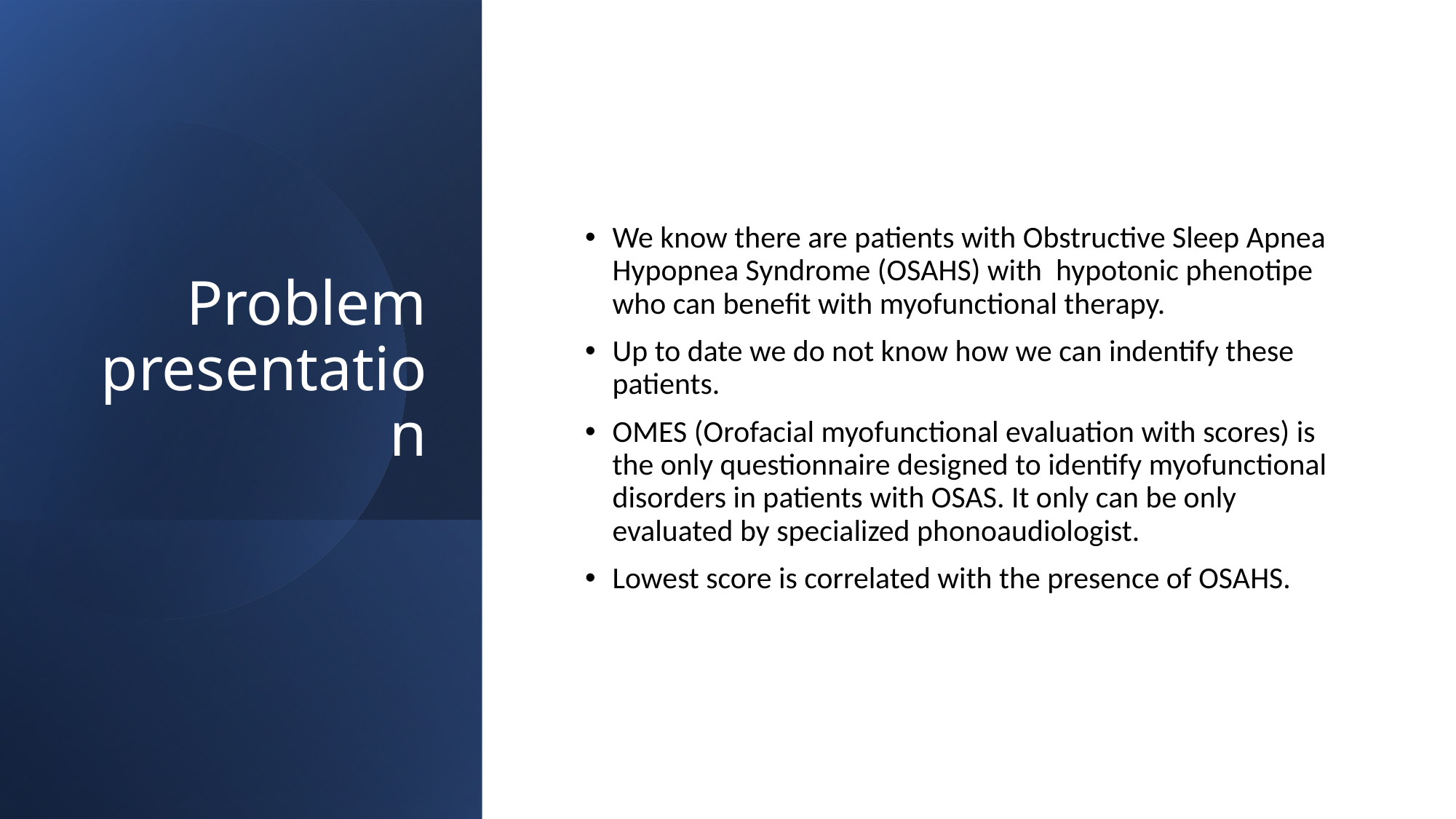

# Problem presentation
We know there are patients with Obstructive Sleep Apnea Hypopnea Syndrome (OSAHS) with hypotonic phenotipe who can benefit with myofunctional therapy.
Up to date we do not know how we can indentify these patients.
OMES (Orofacial myofunctional evaluation with scores) is the only questionnaire designed to identify myofunctional disorders in patients with OSAS. It only can be only evaluated by specialized phonoaudiologist.
Lowest score is correlated with the presence of OSAHS.

## Slide 3
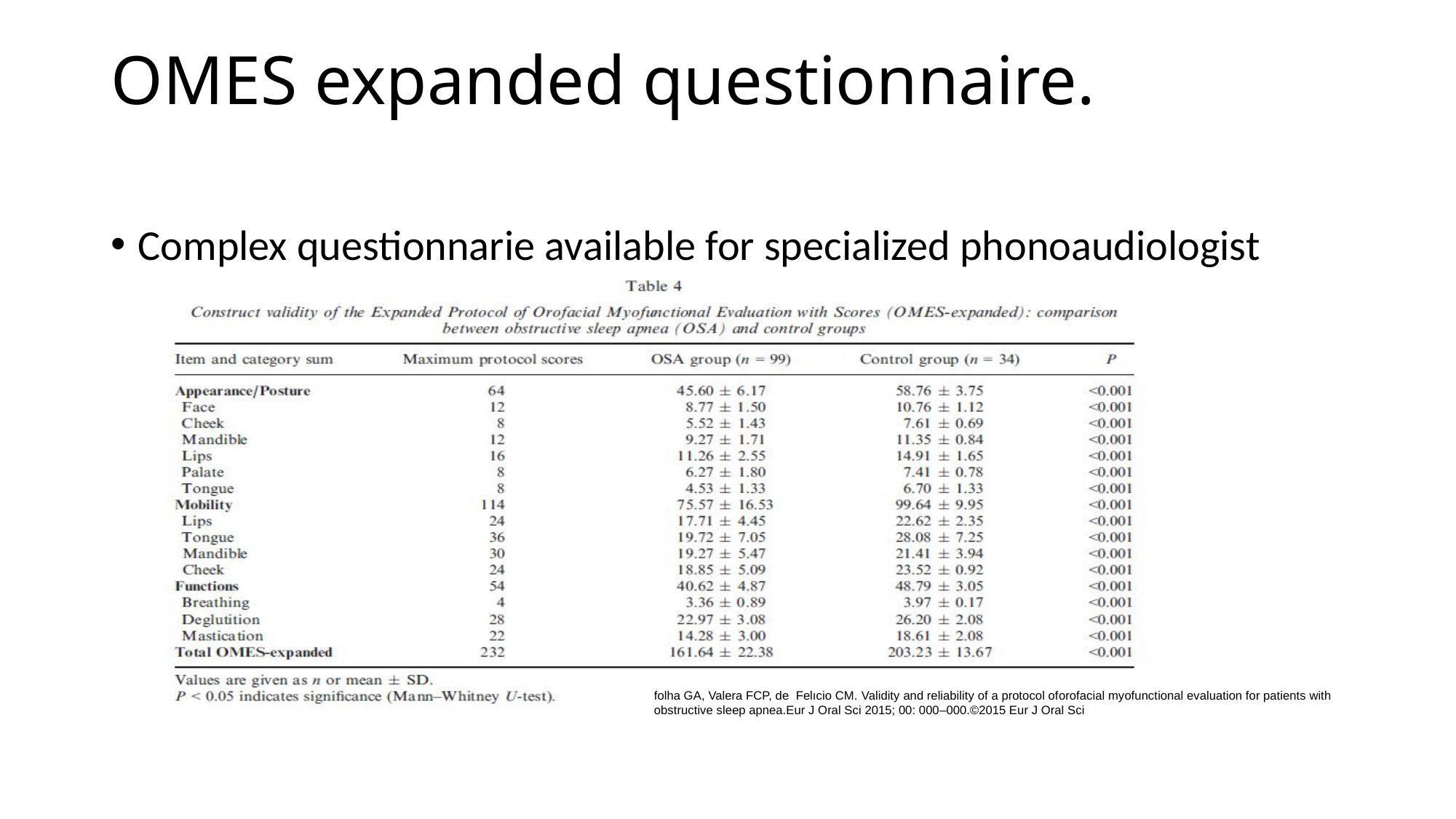

# OMES expanded questionnaire.
Complex questionnarie available for specialized phonoaudiologist
folha GA, Valera FCP, de Felıcio CM. Validity and reliability of a protocol oforofacial myofunctional evaluation for patients with obstructive sleep apnea.Eur J Oral Sci 2015; 00: 000–000.©2015 Eur J Oral Sci

## Slide 4
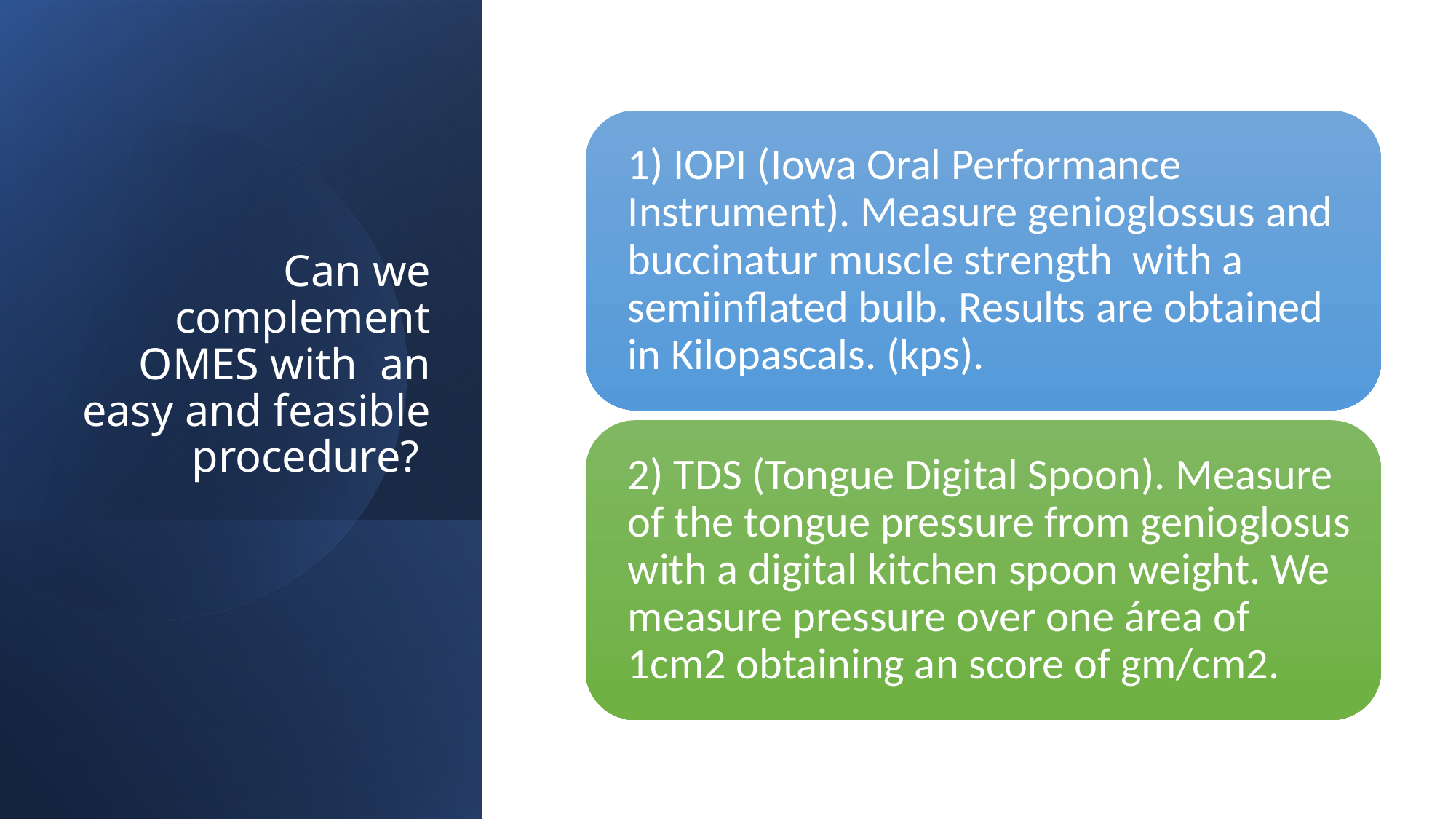

# Can we complement OMES with an easy and feasible procedure?

## Slide 5
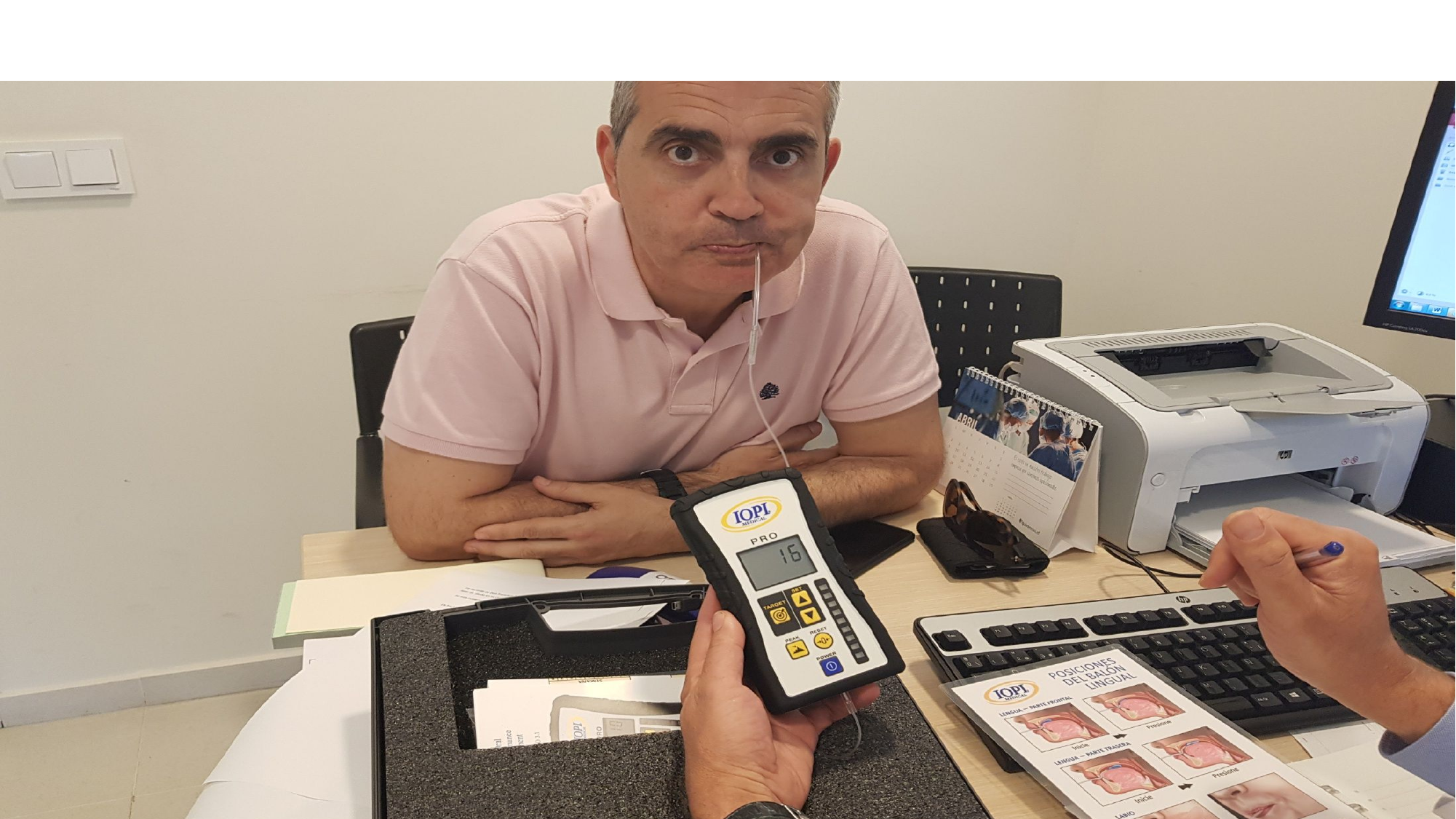

## Slide 6
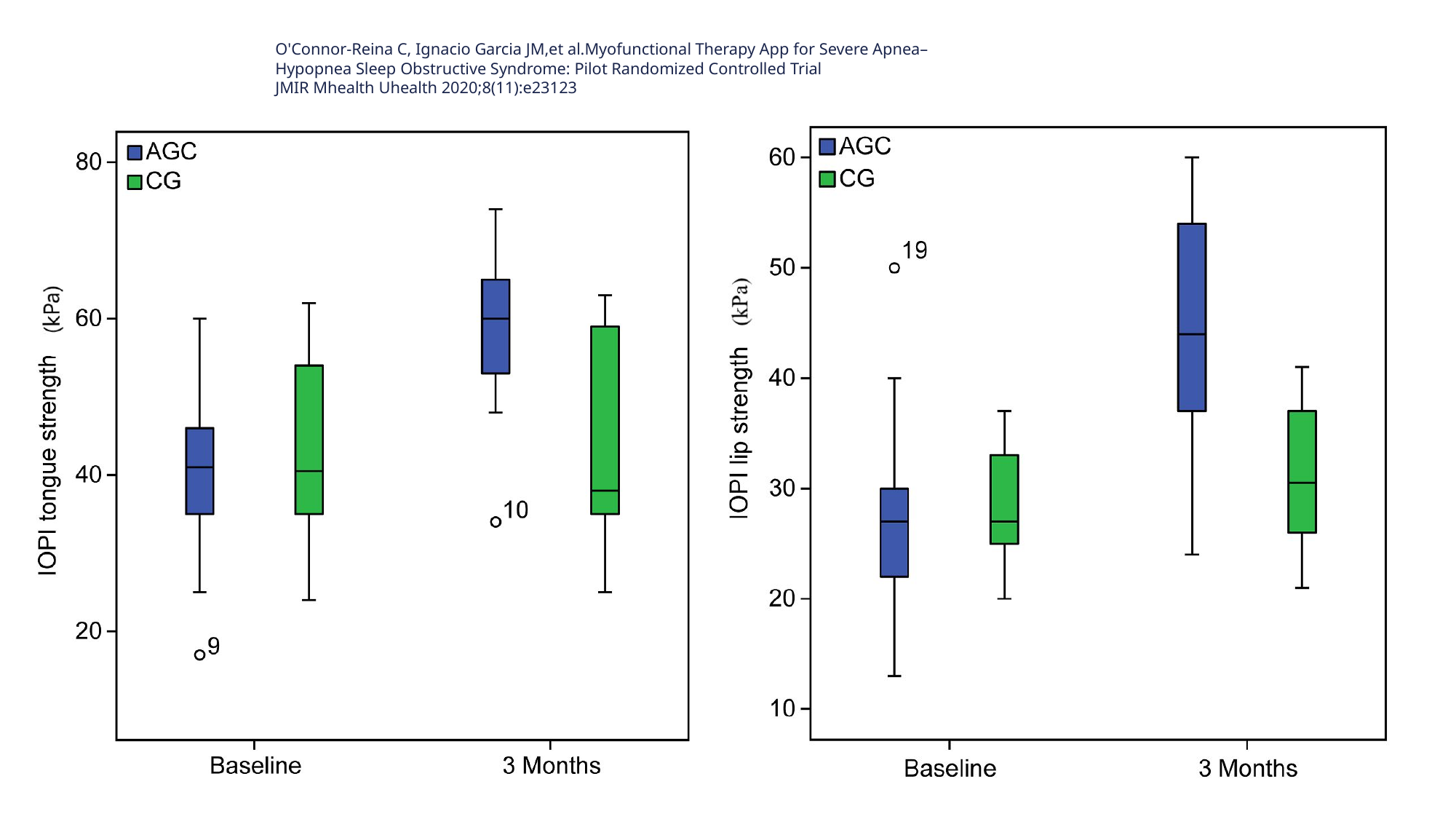

O'Connor-Reina C, Ignacio Garcia JM,et al.Myofunctional Therapy App for Severe Apnea–Hypopnea Sleep Obstructive Syndrome: Pilot Randomized Controlled Trial
JMIR Mhealth Uhealth 2020;8(11):e23123

## Slide 7
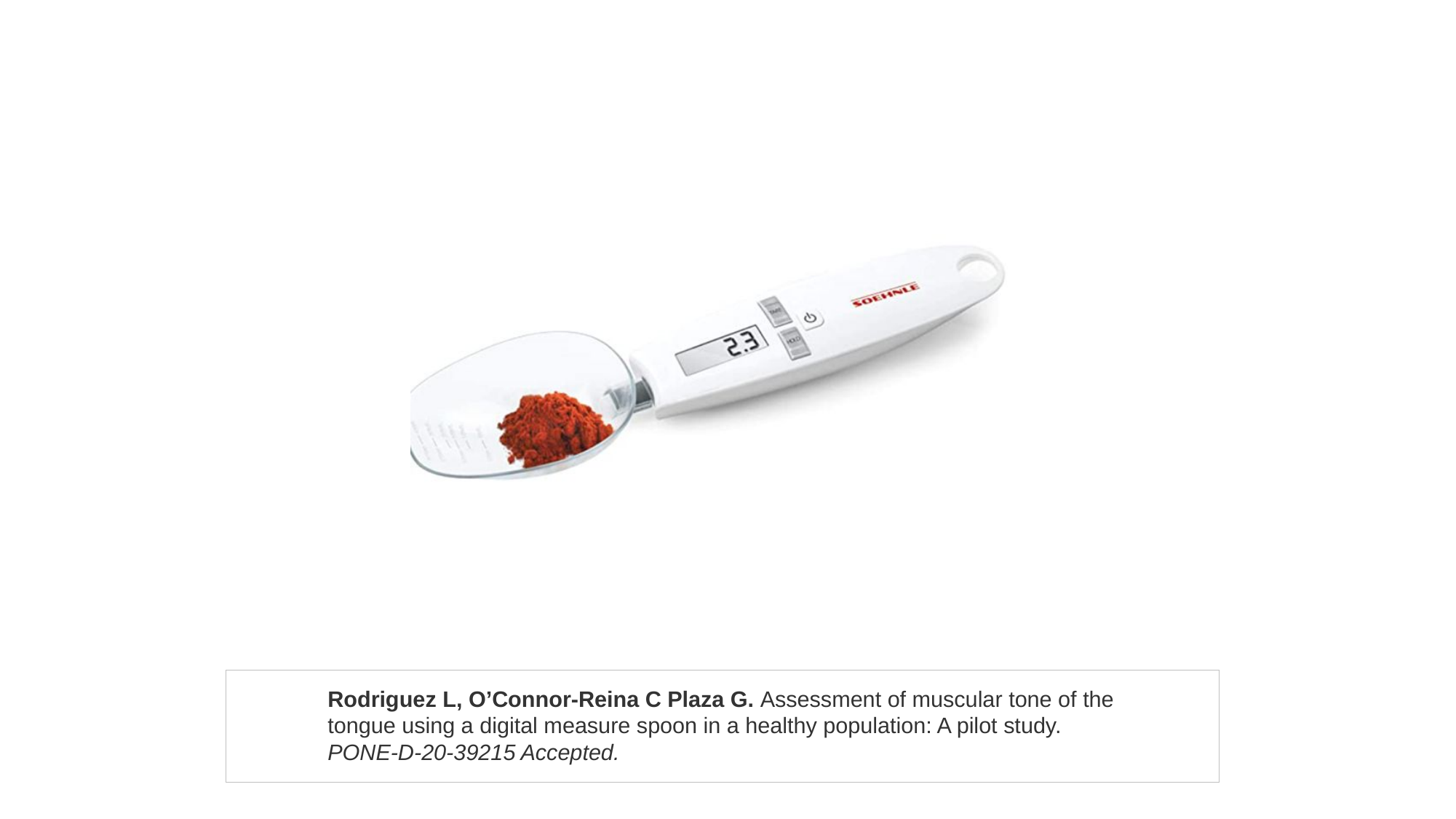

Rodriguez L, O’Connor-Reina C Plaza G. Assessment of muscular tone of the tongue using a digital measure spoon in a healthy population: A pilot study. PONE-D-20-39215 Accepted.

## Slide 8
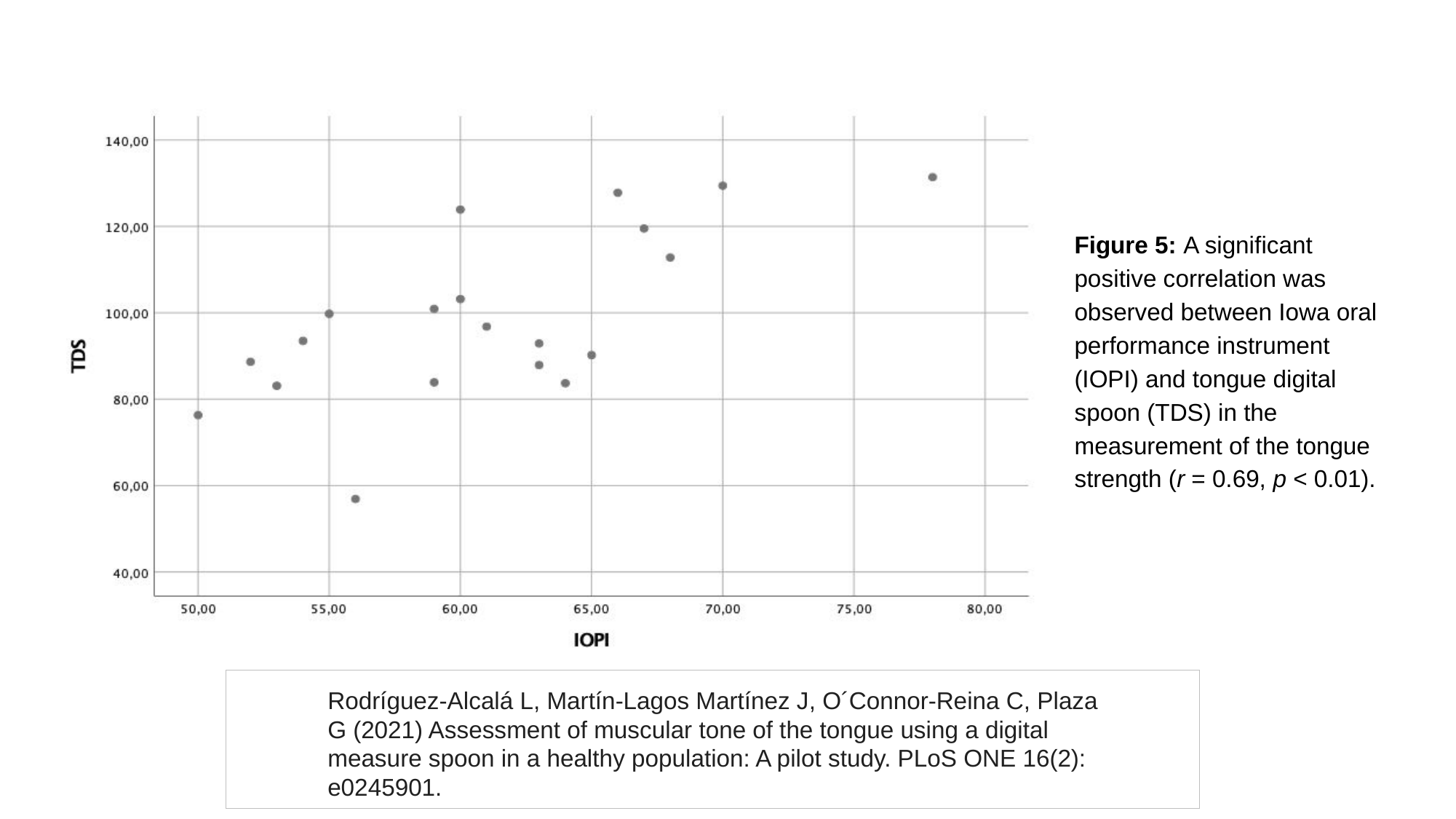

Figure 5: A significant positive correlation was observed between Iowa oral performance instrument (IOPI) and tongue digital spoon (TDS) in the measurement of the tongue strength (r = 0.69, p < 0.01).
Rodríguez-Alcalá L, Martín-Lagos Martínez J, O´Connor-Reina C, Plaza G (2021) Assessment of muscular tone of the tongue using a digital measure spoon in a healthy population: A pilot study. PLoS ONE 16(2): e0245901.

## Slide 9
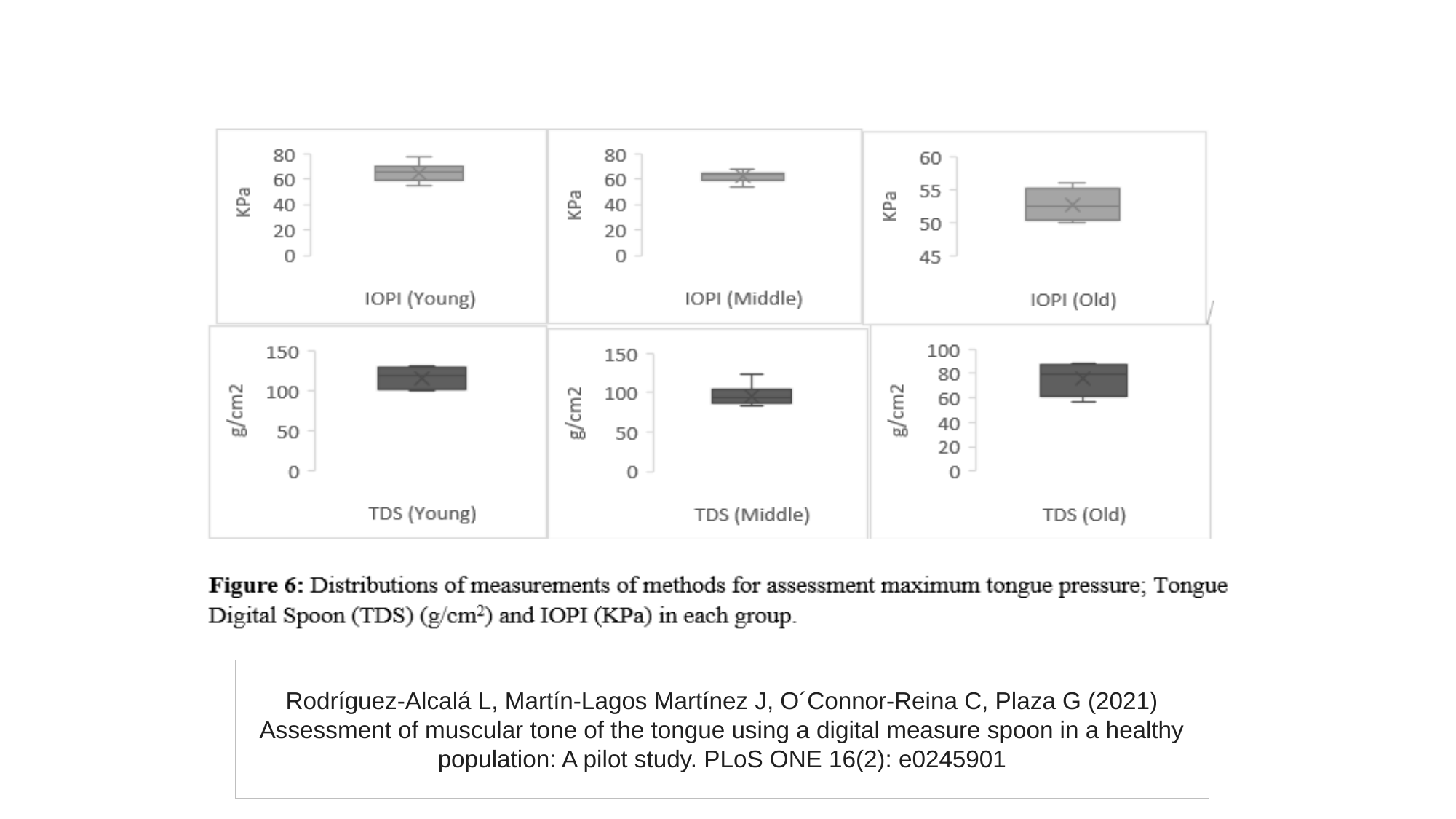

Rodríguez-Alcalá L, Martín-Lagos Martínez J, O´Connor-Reina C, Plaza G (2021) Assessment of muscular tone of the tongue using a digital measure spoon in a healthy population: A pilot study. PLoS ONE 16(2): e0245901

## Slide 10
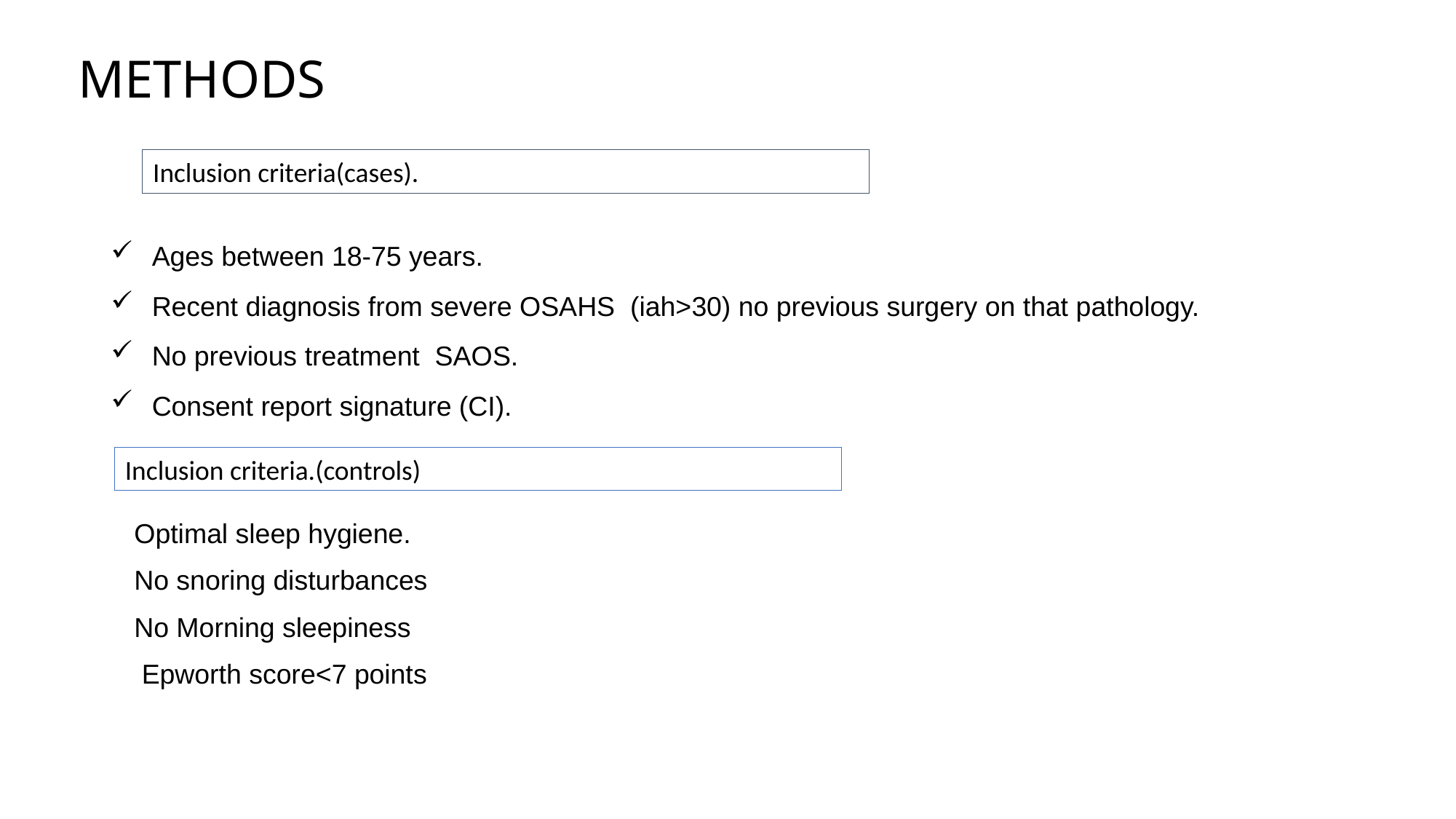

# METHODS
Inclusion criteria(cases).
Ages between 18-75 years.
Recent diagnosis from severe OSAHS (iah>30) no previous surgery on that pathology.
No previous treatment SAOS.
Consent report signature (CI).
Inclusion criteria.(controls)
Optimal sleep hygiene.
No snoring disturbances
No Morning sleepiness
 Epworth score<7 points

## Slide 11
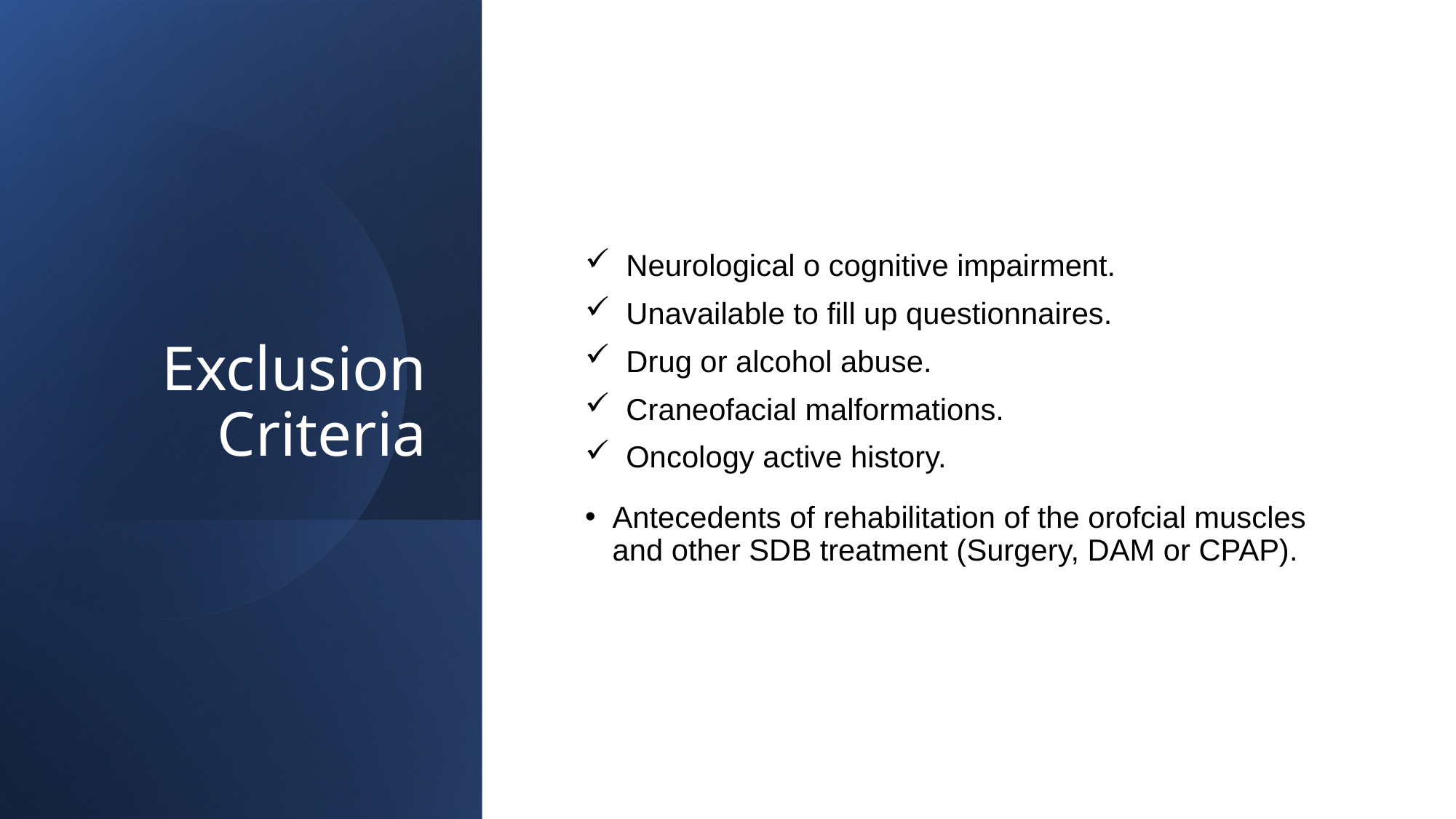

# Exclusion Criteria
Neurological o cognitive impairment.
Unavailable to fill up questionnaires.
Drug or alcohol abuse.
Craneofacial malformations.
Oncology active history.
Antecedents of rehabilitation of the orofcial muscles and other SDB treatment (Surgery, DAM or CPAP).

## Slide 12
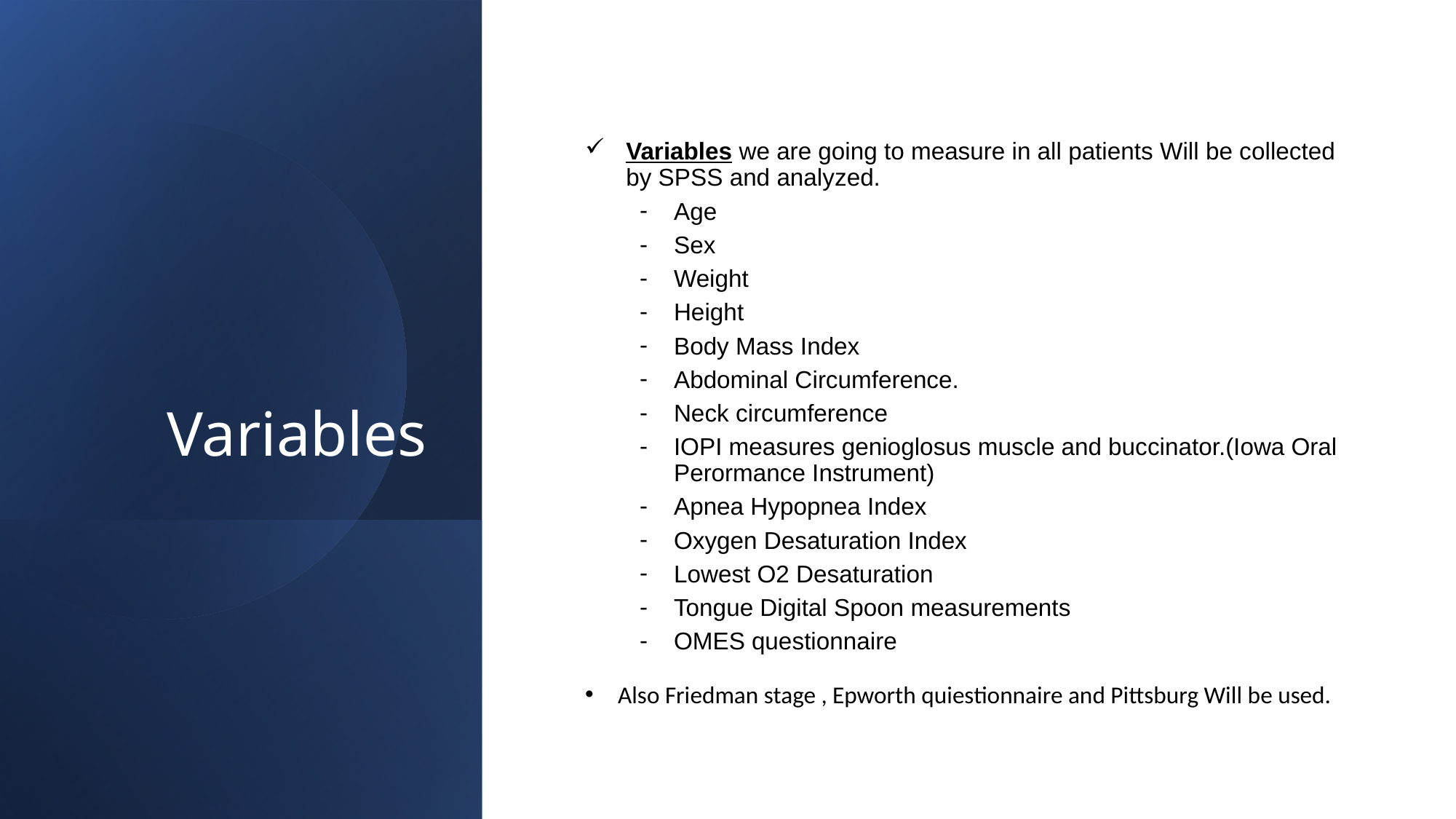

# Variables
Variables we are going to measure in all patients Will be collected by SPSS and analyzed.
Age
Sex
Weight
Height
Body Mass Index
Abdominal Circumference.
Neck circumference
IOPI measures genioglosus muscle and buccinator.(Iowa Oral Perormance Instrument)
Apnea Hypopnea Index
Oxygen Desaturation Index
Lowest O2 Desaturation
Tongue Digital Spoon measurements
OMES questionnaire
 Also Friedman stage , Epworth quiestionnaire and Pittsburg Will be used.

## Slide 13
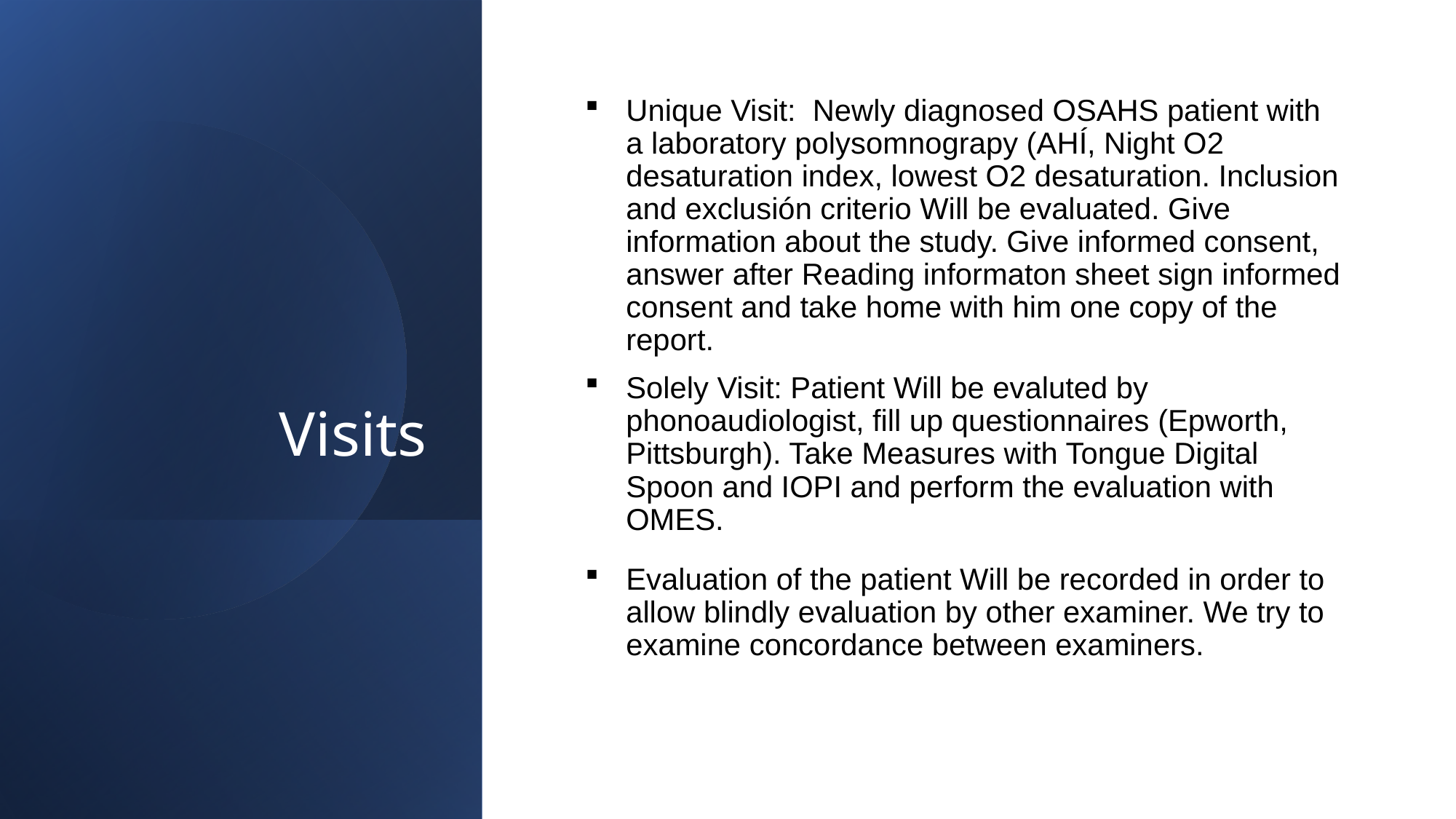

# Visits
Unique Visit: Newly diagnosed OSAHS patient with a laboratory polysomnograpy (AHÍ, Night O2 desaturation index, lowest O2 desaturation. Inclusion and exclusión criterio Will be evaluated. Give information about the study. Give informed consent, answer after Reading informaton sheet sign informed consent and take home with him one copy of the report.
Solely Visit: Patient Will be evaluted by phonoaudiologist, fill up questionnaires (Epworth, Pittsburgh). Take Measures with Tongue Digital Spoon and IOPI and perform the evaluation with OMES.
Evaluation of the patient Will be recorded in order to allow blindly evaluation by other examiner. We try to examine concordance between examiners.

## Slide 14
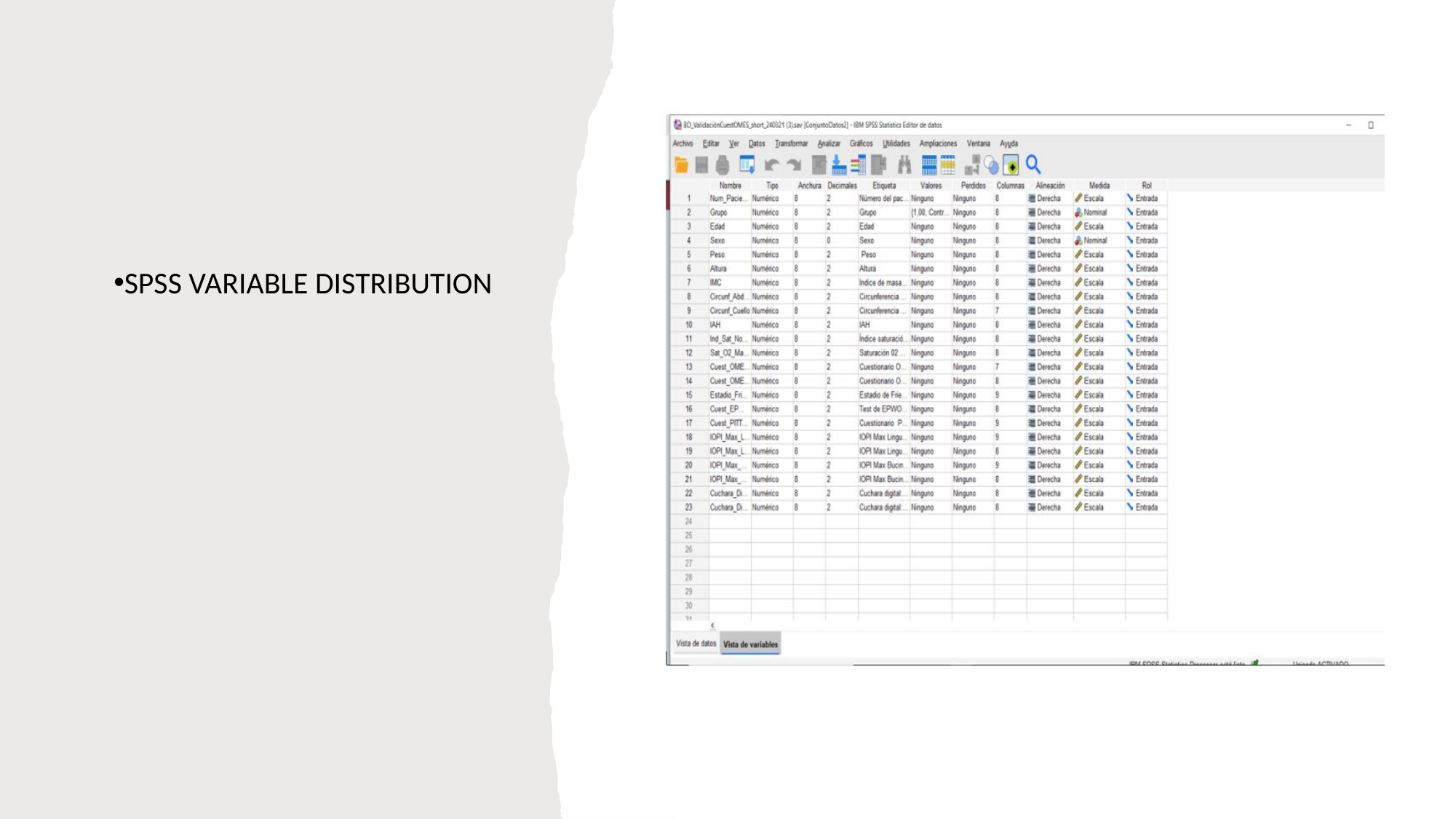

SPSS VARIABLE DISTRIBUTION

## Slide 15
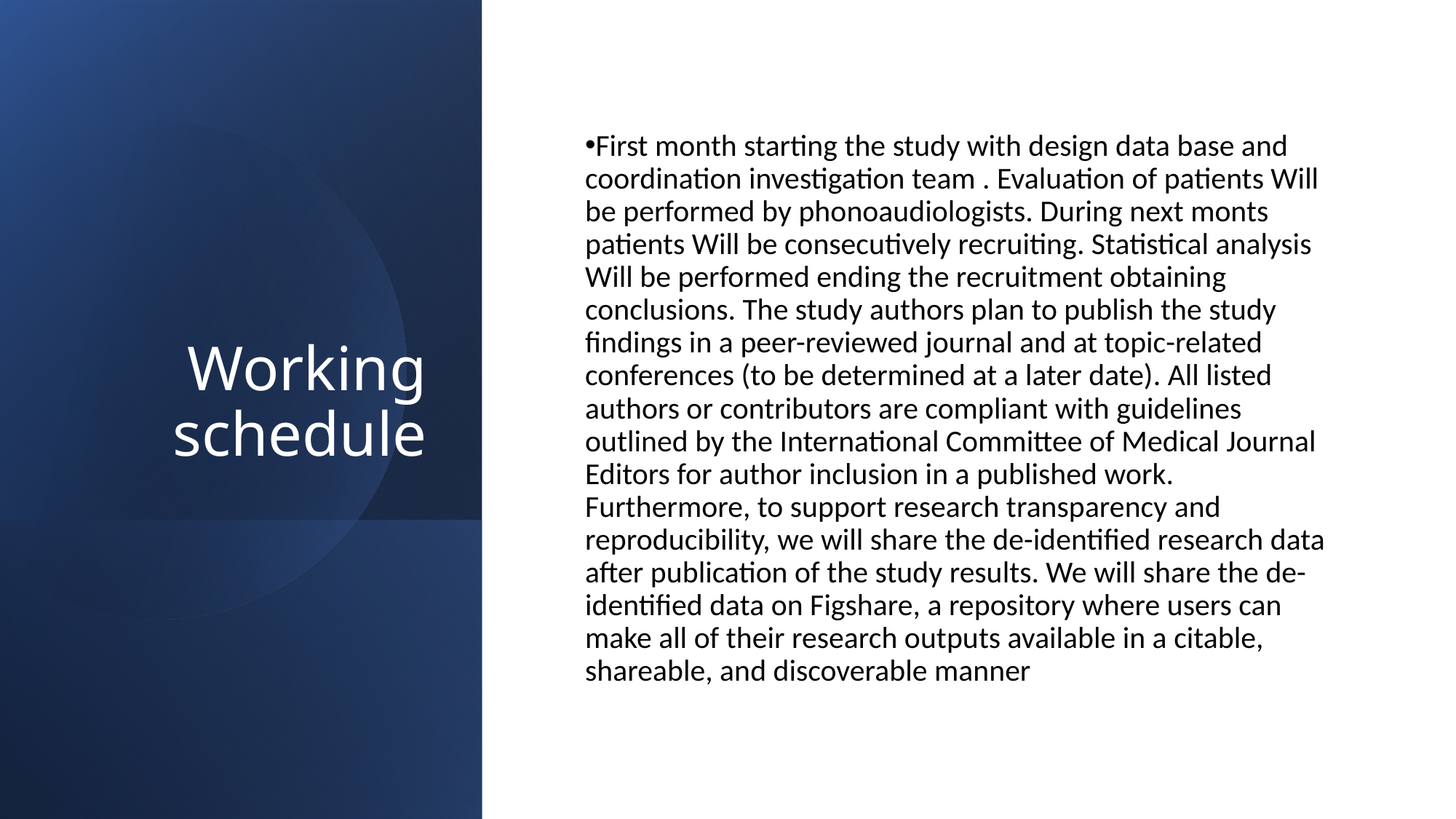

# Working schedule
First month starting the study with design data base and coordination investigation team . Evaluation of patients Will be performed by phonoaudiologists. During next monts patients Will be consecutively recruiting. Statistical analysis Will be performed ending the recruitment obtaining conclusions. The study authors plan to publish the study findings in a peer-reviewed journal and at topic-related conferences (to be determined at a later date). All listed authors or contributors are compliant with guidelines outlined by the International Committee of Medical Journal Editors for author inclusion in a published work. Furthermore, to support research transparency and reproducibility, we will share the de-identified research data after publication of the study results. We will share the de-identified data on Figshare, a repository where users can make all of their research outputs available in a citable, shareable, and discoverable manner

## Slide 16
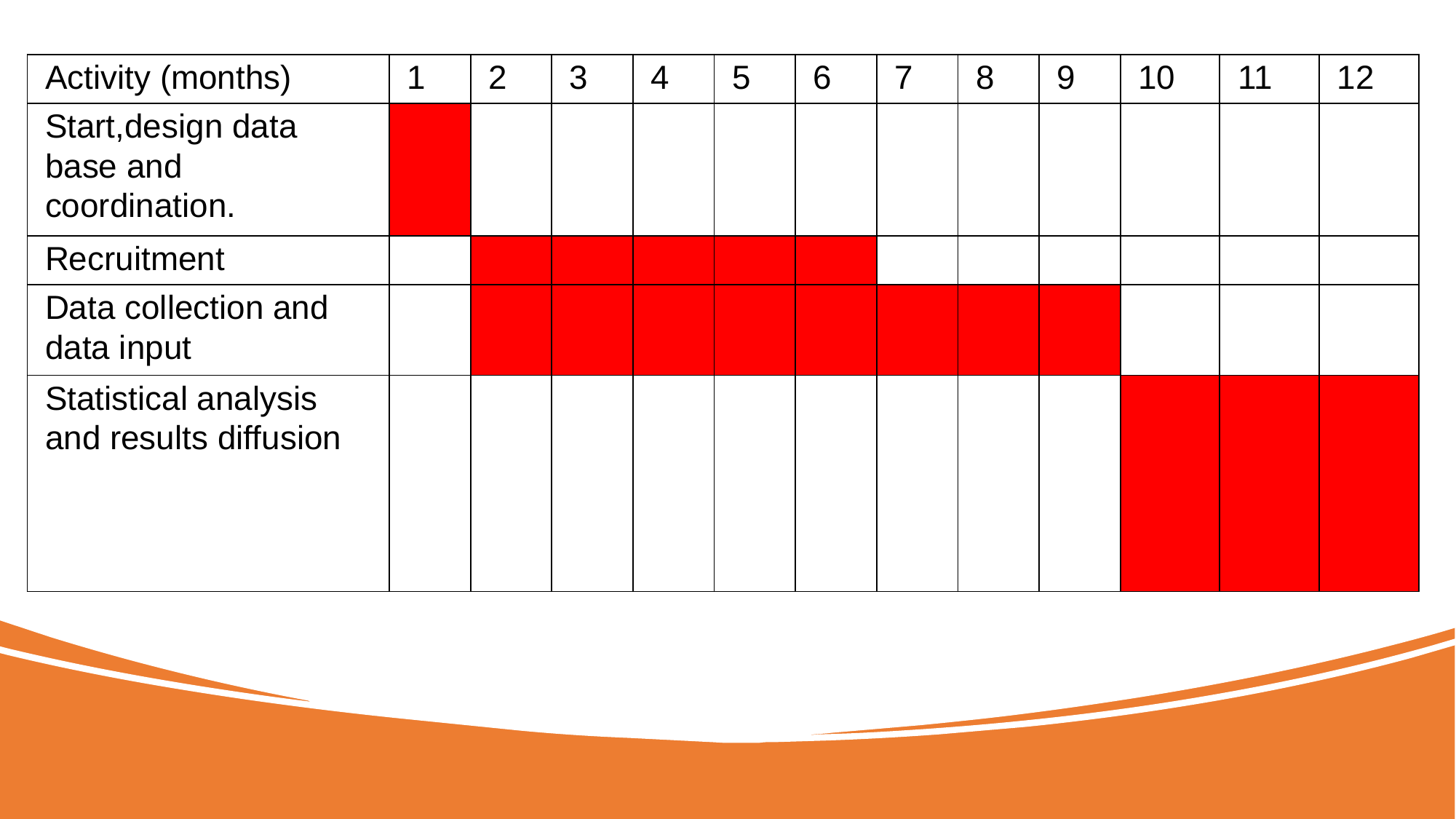

| Activity (months) | 1 | 2 | 3 | 4 | 5 | 6 | 7 | 8 | 9 | 10 | 11 | 12 |
| --- | --- | --- | --- | --- | --- | --- | --- | --- | --- | --- | --- | --- |
| Start,design data base and coordination. | | | | | | | | | | | | |
| Recruitment | | | | | | | | | | | | |
| Data collection and data input | | | | | | | | | | | | |
| Statistical analysis and results diffusion | | | | | | | | | | | | |
