# Supplementary material for: Muscular Assessment in Patients With Severe Obstructive Sleep Apnea Syndrome: Protocol for a Case-Control Study
Source: JMIR Res Protoc. 2021 Aug 6;10(8):e30500. doi: 10.2196/30500 (PMC8380583; doi:10.2196/30500)
Supplement: Multimedia Appendix 2 [file resprot_v10i8e30500_app2.pdf]

Epworth Sleepiness Scale

How likely are you to doze off or fall asleep in the following situations, in contrast to feeling just
tired?
This refers to your usual way of life in recent times.
Even if you haven't done some of these things recently try to work out how they would have affected
you.
Use the following scale to choose the **most appropriate number** for each situation:
0 = would **never** doze
1 = **slight chance** of dozing
2 = **moderate chance** of dozing
3 = **high chance** of dozing
It is important that you answer each question as best you can.
Situation Chance of Dozing (0-3)

1)Sitting and reading ____
2) Watching TV ____
3) Sitting, inactive in a public place (e.g. a theatre or a meeting) _________
4) As a passenger in a car for an hour without a break _________________
5) Lying down to rest in the afternoon when circumstances permit ________
6) Sitting and talking to someone __________________________________
7) Sitting quietly after a lunch without alcohol ________________________
8) In a car, while stopped for a few minutes in the traffic ________________

0-9 Normal
10-24 Refered to specialist
11-15 Mild to moderate risk of sleep apnea
16: High risk of sleep apnea

PATIENT INFORMATION

Please read the following information carefully and ask any questions you may have before signing this informed consent.

Background:

Sleep apnea hypopnea is a significant public health problem in which the airway repetitively closes during sleep. It is associated with snoring, sleep fragmentation, excessive daytime sleepiness and increased cardiovascular risk. There are multiple causes of sleep apnea, and these can include anatomical and physiological factors. The upper airway dilator muscles are crucial for the maintenance of pharyngeal patency and may contribute to the incidence of this disease.

What is the objective/importance of this study?

To study the influence of performing a set of oropharyngeal exercises, known as 'myofunctional therapy', in the progression of patients diagnosed with sleep apnea hypopnea. These exercises are derived from speech therapy and involve exercises using the tongue, soft palate and pharyngeal wall, including sucking, swallowing, chewing, breathing and speaking.

Tongue and oropharynx exercises (myofunctional therapy) may be an attractive alternative treatment for snoring and sleep apnea to improve the quality of life of patients who suffer from this disorder. The problem is that it is currently not known which patients are most suited for this treatment.

Why have you been asked to participate?

You've been asked to participate in this study as you have been diagnosed with sleep apnea, meaning your breathing stops and starts during sleep.

What will your participation involve? What kinds of tests or procedures will you undergo?

Your participation in this study will involve undergoing a functional exploration of your oropharyngeal muscles and you will be asked to complete a series of questionnaires. Measurements will also be taken of the strength of your tongue, your orbicular muscles, as well as your height, weight, BMI and neck and abdominal circumferences. These would be carried out in a single visit to the centre. The second group (the control group) is composed of healthy patients who will also undergo the same examinations. Both groups will be recorded with a camera so that other researchers can evaluate the examinations.

What are the general risks of participating in this study?

So far there have been no complications resulting from this procedure. No additional risk is foreseen for you.

What are the benefits of participating in this study?

To improve the selection of patients who may benefit from myofunctional therapy.

It is highly likely that the results obtained from this study will help to better understand your disease and improve the prognosis and treatment of future patients.

What will happen if I choose to not take part in this study?

Your participation in this study is completely voluntary. In the event that you decide not to participate in the study, this will not affect the treatment and monitoring of your illness by your doctor or the rest of the health personnel who treat your sleep apnea. Likewise, you can withdraw from the study at any time, without having to give a reason.

Who can I contact if I have any questions?

It is important you raise any questions or concerns you may have with the project researchers before signing the consent form. Likewise, you can ask for additional information about the study and what will happen throughout it by contacting the main project researcher, Dr Carlos O Connor (658059669) or by email coconnor@us.es, or the pneumology service via telephone 645 802 433 or email pulmonología.hmb@quiron.es

Confidentiality:

All your data, as well as all medical information related to your illness, will be treated with absolute confidentiality by the personnel in charge of the research. Likewise, if the results of the study are published in scientific journals, the personal data of the patients who took part in the project will not be shared at any point.

As provided by Organic Law 15/1999 on the Protection of Personal Data, you may exercise your right to access, rectify and delete your data by contacting the main project researcher.

Patient involvement:

- Participation is completely voluntary.
- The patient can withdraw from the study at any time, without giving a reason and without this affecting their medical care.
- All personal data obtained from this study is confidential and will be treated in accordance with the Organic Law on the Protection of Personal Data 15/99.
- The information obtained will be used exclusively for the specific purposes of this study.

INFORMED CONSENT – PATIENT WRITTEN CONSENT

VALIDATION OF THE OMES PROTOCOL AGAINST THE OBJECTIVE MEASUREMENT OF OROPHARYNGEAL MUSCLE TONE USING THE IOPI AND THE TONGUE DIGITAL SPOON

I, (Name(s) and surname(s)):...

- Have read and I understand the information provided with this consent form (in the Patient Information).

I have had the opportunity to ask questions about the study: VALIDATION OF THE OMES QUESTIONNAIRE AGAINST OBJECTIVE MEASUREMENT OF OROPHARYNGEAL MUSCLE TONE USING THE IOPI AND THE DIGITAL SPOON

-

I have received enough information about the study VALIDATION OF THE OMES QUESTIONNAIRE AGAINST OBJECTIVE MEASUREMENT OF OROPHARYNGEAL MUSCLE TONE USING THE IOPI AND THE DIGITAL SPOON

- I have spoken with the reporting healthcare professional: …………………………………………………………

- I understand that my participation is voluntary and I that I do not have to take part.

- I have been informed that all the data obtained from this study will be confidential and will be treated in accordance with the Organic Law on the Protection of Personal Data 15/99.

- I have been informed that the information obtained will only be used for the specific purposes of the study.

Yes No

I understand that I can withdraw from the study:

- Whenever I want.
- Without having to give a reason.
- With no impact on my medical care.

I freely agree to participate in the *project entitled* VALIDATION OF THE OMES QUESTIONNAIRE IN FRONT OF OBJECTIVE MEASUREMENT OF OROPHARYNGEAL MUSCLE TONE USING THE IOPI AND THE DIGITAL SPOON

Patient's signature
(or legal representative, if applicable)

Reporting healthcare
professional's signature

Name(s) and surname(s):……………….
Date: ………………………………

Name(s) and surname(s): ………..
Date: ……………………….

WITHDRAWAL OF CONSENT

I hereby REVOKE any authorisations set forth in this document, which is without effect from the moment of signature.

The consequences that this revocation could have on the progression of my process have been explained to me, and I understand and accept them.

In………………., on…….…………………………. 20..

Patient's signature
(or legal representative, if applicable)

Name(s) and surname(s):……………….

PITTSBURGH SLEEP QUALITY INDEX (PSQI)

INSTRUCTIONS: The following questions relate to your usual sleep habits during the past month only. Your answers should indicate the most accurate reply for the majority of days and nights in the past month. Please answer all questions.

1. During the past month, when have you usually gone to bed at night?

   USUAL BED TIME ____________________

2. During the past month, how long (in minutes) has it usually take you to fall asleep each night?

   NUMBER OF MINUTES ____________________

3. During the past month, when have you usually gotten up in the morning?

   USUAL GETTING UP TIME ____________________

4. During the past month, how many hours of actual sleep did you get at night? (This may be different than the number of hours you spend in bed.)

   HOURS OF SLEEP PER NIGHT ____________________

INSTRUCTIONS: For each of the remaining questions, check the one best response. Please answer all questions.

5. During the past month, how often have you had trouble sleeping because you...

	Not during the past month	Less than once a week	Once or twice a week	Three or more times a week
(a) ...cannot get to sleep within 30 minutes	☐	☐	☐	☐
(b) ...wake up in the middle of the night or early morning	☐	☐	☐	☐
(c) ...have to get up to use the bathroom	☐	☐	☐	☐
(d ...cannot breathe comfortably	☐	☐	☐	☐
(e) ...cough or snore loudly	☐	☐	☐	☐
(f) ...feel too cold	☐	☐	☐	☐
(g) ...feel too hot	☐	☐	☐	☐
(h) ...had bad dreams	☐	☐	☐	☐
(i) ...have pain	☐	☐	☐	☐

(j) Other reason(s), please describe

____

____

How often during the past month have you had trouble sleeping because of this?	☐	☐	☐	☐

	Very good	Fairly good	Fairly bad	very bad
6. During the past month, how would you rate your sleep quality overall?	☐	☐	☐	☐

	Not during the past month	Less than once a week	Once or twice a week	Three or more times a week
7. During the past month, how often have you taken medicine (prescribed or "over the counter") to help you sleep?	☐	☐	☐	☐
8. During the past month, how often have you had trouble staying awake while driving, eating meals, or engaging in social activity?	☐	☐	☐	☐

	No problem at all	Only a very slight problem	Somewhat of a problem	A very big problem
9. During the past month, how much of a problem has it been for you to keep up enough enthusiasm to get things done?	☐	☐	☐	☐

	No bed partner or roommate	Partner/ roommate in other room	Partner in same room, but not same bed	Partner in same bed
10. During the past month, how much of a problem has it been for you to keep up enough enthusiasm to get things done?	☐	☐	☐	☐

If you have a roommate or bed partner, ask him/her how often in the past month you have had...

	Not during the past month	Less than once a week	Once or twice a week	Three or more times a week
(a) ...loud snoring	☐	☐	☐	☐
(b) ...long pauses between breaths while asleep	☐	☐	☐	☐
(c) ...legs twitching or jerking while you sleep	☐	☐	☐	☐
(d) ...episodes of disorientation or confusion during sleep	☐	☐	☐	☐
(e) Other restlessness while you sleep; please describe	☐	☐	☐	☐

____

____

SCORING INSTRUCTIONS FOR THE PITTSBURGH SLEEP QUALITY INDEX:

The Pittsburgh Sleep Quality Index (PSQI) contains 19 self-rated questions and 5 questions rated by the bed partner or roommate (if one is available). Only self-rated questions are included in the scoring. The 19 self-rated items are combined to form seven "component" scores, each of which has a range of 0-3 points. In all cases, a score of "0" indicates no difficulty, while a score of "3" indicates severe difficulty. The seven component scores are then added to yield one "global" score, with a range of 0-21 points, "0" indicating no difficulty and "21 " indicating severe difficulties in all areas.

Scoring proceeds as follows:

Component 1: Subjective sleep quality

Examine question #6, and assign scores as follows:

Response	Component 1 score
"Very good"	0
"Fairly good"	1
"Fairly bad"	2
"Very bad"	3

Component 1 score: ________

Component 2: Sleep latency

1. Examine question #2, and assign scores as follows:

Respo\nse	Score
≤15 minutes	0
16-30 minutes	1
31-60 minutes	2
> 60 minutes	3
Question #2 score:	________

2. Examine question #5a, and assign scores as follows:

Response	Score
Not during the past month	0
Less than once a week	1
Once or twice a week	2
Three or more times a week	3
Question #5a score:	________

3. Add #2 score and #5a score

Sum of #2 and #5a: ________

4. Assign component 2 score as follows:

Sum of #2 and #5a	Component 2 score
0	0
1-2	1
3-4	2
5-6	3

Component 2 score: ________

Component 3: Sleep duration

Examine question #4, and assign scores as follows:

Response	Component 3 score
> 7 hours	0
6-7 hours	1
5-6 hours	2
< 5 hours	3

Component 3 score:________

Component 4: Habitual sleep efficiency

1. Write the number of hours slept (question #4) here:________
2. Calculate the number of hours spent in bed:

   Getting up time (question #3):________

   Bedtime (question #1):________

   ____

   Number of hours spent in bed:________

3. Calculate habitual sleep efficiency as follows:

   (Number of hours slept/Number of hours spent in bed) X 100 = Habitual sleep efficiency (%)

   (________/________) X 100 = %

4. Assign component 4 score as follows:

Habitual sleep efficiency %	Component 4 score
> 85%	0
75-84%	1
65-74%	2
< 65%	3

Component 4 score:________

Component 5: Step disturbances

1. Examine questions #5b-5j, and assign scores for each question as follows:

Response	Score
Not during the past month	0
Less than once a week	1
Once or twice a week	2
Three or more times a week	3

5b score: ________

5c score: ________

5d score: ________

5e score: ________

5f score: ________

5g score: ________

5h score: ________

5i score: ________

5j score: ________

2. Add the scores for questions #5b-5j:

Sum of #5b-5j: ________

3. Assign component 5 score as follows:

Sum of #5b-5j	Component 5 score
0	0
1-9	1
10-18-4	2
19-27	3

Component 5 score: ________

Component 6: Use of sleeping medication

Examine question #7 and assign scores as follows:

Response	Component 6 score
Not during the past month	0
Less than once a week	1
Once or twice a week	2
Three or more times a week	3

Component 6 score: ________

Component 7: Daytime dysfunction

1. Examine question #8, and assign scores as follows:

Response	Score
Never	0
Once or twice	1
Once or twice each week	2
Three or more times each week	3

Question#8 score: ________

2. Examine question #9, and assign scores as follows:

Response	Score
No problem at all	0
Only a very slight problem	1
Somewhat of a problem	2
A very big problem	3

Question #9 score: ________

3. Add the scores for question #8 and #9:

Sum of #8 and #9: ________

4. Assign component 7 score as follows:

Sum of #8 and #9	Component 7 score
0	0
1-2	1
3-4	2
5-6	3

Component 7 score: ________

Global PSQI Score

Add the seven component scores together:

Global PSQI Score: ________
