# Supplementary material for: Muscular Assessment in Patients With Severe Obstructive Sleep Apnea Syndrome: Protocol for a Case-Control Study
Source: JMIR Res Protoc. 2021 Aug 6;10(8):e30500. doi: 10.2196/30500 (PMC8380583; doi:10.2196/30500)
Supplement: Multimedia Appendix 3 [file resprot_v10i8e30500_app3.pdf]

Appearance and posture.

Face				Scores
Symmetry between right and left side	Normal			(4)
Asymmetry	Light dysfunction			(3)
	Moderate dysfunction			(2)
	Severe dysfunction			(1)
Increased side	Right	Left		
Proportion between thirds of the face	Normal			(4)
Altered proportion	Light dysfunction			(3)
	Moderate dysfunction			(2)
	Severe dysfunction			(1)
Increased third of the face	Inferior	Middle	Superior	
Nasolabial sulcus	Normal for age			(4)
Marked nasolabial sulcus	Light dysfunction			(3)
	Moderate dysfunction			(2)
	Severe dysfunction			(1)
Result				
Maximum score = 12				

Cheek appearance				Scores
Volume	Normal			(4)
Increased volume	Light dysfunction			(3)
	Moderate dysfunction			(2)
	Severe dysfunction			(1)
Increased side	Right	Left	Right and left	
Tension/configuration	Normal			(4)
Flaccid/drooping	Light dysfunction			(3)
	Moderate dysfunction			(2)
	Severe dysfunction			(1)
Result				
Maximum score = 08				

Mandible/maxilla relation				Scores
Vertical mandibular posture—with free way space		Normal posture		(4)
Altered vertical relation—without free way space				
Occlusion of the teeth	Without apparent tension	Light dysfunction		(3)
	Apparent tension	Moderate dysfunction		(2)
	Apparent tension	Severe dysfunction		(1)
Open mouth—exceeds the free way space (more than 4 mm)		Light dysfunction		(3)
		Moderate dysfunction		(2)
		Severe dysfunction		(1)
Anteroposterior relation		Normal		(4)
Altered anteroposterior relation		Light dysfunction		(3)
		Moderate dysfunction		(2)
		Severe dysfunction		(1)
Overjet		Positive	Negative	
Relation with the midline		Normal		(4)
Altered (lateral deviation)		Light dysfunction		(3)
		Moderate dysfunction		(2)
		Severe dysfunction		(1)
Deviation side		To right	To left	
Result				
Maximum score = 12				

Lips		Scores
Resting lips function		
Normal lips closure	Normal lips function	(4)
Dysfunction		
Lips closure with effort (Presence of lips function, but with increased activity of lips and *mentalis* muscle)	Light dysfunction	(3)
	Moderate dysfunction	(2)
	Severe dysfunction	(1)

Lips				Scores
Absence of lips closure	Light dysfunction (half-open)			(3)
	Moderate dysfunction			(2)
	Severe dysfunction			(1)
Volume and configuration	Normal			(4)
Reduced volume and stretched	Light dysfunction			(3)
	Moderate dysfunction			(2)
	Severe dysfunction			(1)
Increased volume	Light dysfunction			(3)
	Moderate dysfunction			(2)
	Severe dysfunction			(1)
Labial commissures				
At the level of the rima of the mouth and symmetry between sides	Normal			(4)
Below of the rima of the mouth (depressed) and/or asymmetrics	Light dysfunction			(3)
	Moderate dysfunction			(2)
	Severe dysfunction			(1)
Side below the rima of the mouth	Right	Left	Both	
Result				
Maximum score = 12				

Mentalis muscle		Scores
Contraction not apparent (with lips closure)	Normal	(4)
Increased activity	Light dysfunction	(3)
	Moderate dysfunction	(2)
	Severe dysfunction	(1)
Result		
Maximum score = 4		

Tongue				Scores
Position/appearance				
Contained in the oral cavity	Normal			(4)
Compressed by tense dental occlusion	Clenching			(3)
Compressed and with marks	Clenching			(2)
Between dental arches (or margins)				
	At limit of the incisal surfaces, with reduced vertical dimension of occlusion (VDO)			(3)
	At limit of the incisal surfaces or on the floor of mouth, with normal free way space			(2)
	Exceeds the incisal surfaces/or vestibular cusps			(1)
Between the dental arches, when present overbite or overjet	At limit of the incisal surfaces			(3)
	Exceeds the incisal surfaces			(2)
	Greatly exceeds the incisal and/or vestibular surfaces			(1)
Local of the interposition	Right	Left	Both	
	Anterior	Posterior	Total	
Appearance/volume				
Volume compatible with the oral cavity	Normal			(4)
Volume increased and/or widened	Light dysfunction			(3)
	Moderate dysfunction			(2)
	Severe dysfunction			(1)
Result				
Maximum score = 8				

Palate appearance		Scores
Width	Normal	(4)
Decreased width (narrow)	Light dysfunction	(3)
	Moderate dysfunction	(2)
	Severe dysfunction	(1)
Height	Normal	(4)
Increased height (deep)	Light dysfunction	(3)
	Moderate dysfunction	(2)
	Severe dysfunction	(1)
Result		
Maximum score = 08		

Mobility.

Performance	Lip movements				
	Protrusion	Retrusion	Lateral to right	Lateral to left	Scores
Normal	(6)	(6)	(6)	(6)	
Insufficient ability	(5)	(5)	(5)	(5)	
Insufficient ability with associated movements	(4)	(4)	(4)	(4)	
Insufficient ability with tremor	(3)	(3)	(3)	(3)	

Performance	Lip movements				
	Protrusion	Retrusion	Lateral to right	Lateral to left	Scores
Insufficient ability with associated movements and tremor	(2)	(2)	(2)	(2)	
Absence of ability (does not perform)	(1)	(1)	(1)	(1)	
Result (Sum)					
Maximum sum = 24					

Performance	Tongue movements						
	Protrusion	Retrusion	Lateral to right	Lateral to left	Raising	Lowering	Scores
Normal	(6)	(6)	(6)	(6)	(6)	(6)	
Insufficient ability	(5)	(5)	(5)	(5)	(5)	(5)	
Insufficient ability with associated movements	(4)	(4)	(4)	(4)	(4)	(4)	
Insufficient ability with tremor	(3)	(3)	(3)	(3)	(3)	(3)	
Insufficient ability with associated movements and tremor	(2)	(2)	(2)	(2)	(2)	(2)	
Absence of ability (does not perform)	(1)	(1)	(1)	(1)	(1)	(1)	
Result (Sum)							
Maximum sum = 36							

Performance	Cheek movements				
	To inflate	To suck	To retract	To transfer the air from right to left	Scores
Normal	(6)	(6)	(6)	(6)	
Insufficient ability	(5)	(5)	(5)	(5)	
Insufficient ability with associated movements	(4)	(4)	(4)	(4)	
Insufficient ability with tremor	(3)	(3)	(3)	(3)	
Insufficient ability with associated movements and tremor	(2)	(2)	(2)	(2)	
Absence of ability (does not perform)	(1)	(1)	(1)	(1)	
Result (Sum)					
Maximum sum = 24					

Performance	Jaw movements					
	Opening	Closing	Right laterality	Left laterality	Protrusion	Scores
Normal	(6)	(6)	(6)	(6)	(6)	
Insufficient ability	(5)	(5)	(5)	(5)	(5)	
Insufficient ability with associated movements	(4)	(4)	(4)	(4)	(4)	
Insufficient ability with deviations	(3)	(3)	(3)	(3)	(3)	
Insufficient ability with associated movements and deviations	(2)	(2)	(2)	(2)	(2)	
Absence of ability (does not perform)	(1)	(1)	(1)	(1)	(1)	
Result (Sum)						
Maximum sum = 30						

Functions.

Breathing: mode		Scores
Nasal breathing	Normal	(4)
Mouth breathing	Light dysfunction	(3)
	Moderate dysfunction	(2)
	Severe dysfunction	(1)
Result		

Deglutition: lips behavior		Scores
Lips closure without effort	Normal	(6)
Lips closure with effort or with tongue between dental arches	Light dysfunction	(4)
	Moderate dysfunction	(3)
	Severe dysfunction	(2)
Absence of lips closure	Does not perform the function	(1)
Result		

Deglutition: tongue behavior				Scores
Contained in the oral cavity	Normal			(4)
Between dental arches (or alveolar margins)	At limit of the incisal surfaces, with reduced VDO			(3)
	At limit of the incisal surfaces, with normal VDO			(2)
	Exceeds the incisal surfaces and/or vestibular cusps			(1)
Interposed with the teeth, when present overbite or overjet	At limit of the incisal surfaces			(3)
	Exceeds the incisal surfaces and/or vestibular cusps			(2)
	Greatly exceeds the incisal surfaces or vestibular cusps			(1)
Local interposition	Right	Left	Both	
	Anterior	Posterior	Total	
Result				
Maximum score = 10				

Deglutition: other behaviors and change signs	Scores	
	Present	Absent
Movements of the head or of other parts of the body	(1)	(2)
Mandible sliding	(1)	(2)
Facial muscle tension	(1)	(2)
Food escape	(1)	(2)
Choking	(1)	(2)
Noise	(1)	(2)
Result		

Deglutition: efficiency	Scores
Solidy bolus	
No more than one repetition of the deglutition	(3)
Two or three repetitions	(2)
Multiple deglutitions	(1)
Liquid bolus	
No more than one repetition of the deglutition	(3)
Two or three repetitions	(2)
Multiple deglutitions	(1)
Result	
Total deglutition result	

Mastication: bite		Scores
Incisors	Normal	(4)
Canines-premolars		(3)
Molars		(2)
Does not bite		(1)
Result		

Mastication: type			Scores
Bilateral	Alternated (50%/50% to 40%/60%)		(10)
	Simultaneous (vertical)		(8)
Unilateral	Preference – grade 1 – (61–77%)		(6)
	Preference – grade 2 – (78–94%)		(4)
	Chronic (95–100%)		(2)
Preferred side	Right	Left	
Anterior	Trituration on the incisors		(2)
Does not perform the function	Does not triturate		(1)
Result			

Mastication: other behaviors and change signs	Scores	
	Present	Absent
Movements of the head or of other parts of the body	(1)	(2)
Altered posture of the head or of other parts of the body	(1)	(2)
Food escape	(1)	(2)
Result		

Total mastication result
Time spent to ingest food =
